# Loneliness without an epidemic: gendered pathways, health consequences, and intervention gaps among men in the United States

**DOI:** 10.3389/fpubh.2026.1817065

**Published:** 2026-06-02

**Authors:** Dalia Chowdhury, Ethan Corbett, Jerry Laney, Skylar Van Winkle, Alina Amjad Khan, Erica Edmonds, Matthew Evan Sprong

**Affiliations:** 1 Department of Rehabilitation and Health Services, University of North Texas, Denton, TX, United States; 2 Department of Psychology, University of North Texas, Denton, TX, United States; 3 Sante Center for Healing, Argyle, TX, United States; 4 Department of Addictions Studies & Behavioral Health, Governors State University, University Park, IL, United States

**Keywords:** cardiovascular risk, gender-responsive interventions, loneliness, masculinity norms, men’s mental health, social isolation

## Abstract

**Introduction:**

Male loneliness has become one of the most significant public health concerns in recent years, also referred to as an “epidemic.” Research demonstrates that men are at greater risk than women for severe health impacts resulting from loneliness; therefore, a gender-informed synthesis of patterns, mechanisms, and intervention gaps is needed to provide clarity.

**Purpose:**

This systematic review evaluates the current literature on loneliness among men in the United States. Specifically, this evaluation examines prevalence and temporal trends, gendered and life-course risk factors, health consequences, and the effectiveness of extant interventions addressing loneliness.

**Methodology:**

Following PRISMA recommendations, we searched PubMed/MEDLINE, CINAHL, PsycINFO, SCOPUS, and Google Scholar for peer-reviewed empirical studies and systematic reviews that included male-specific data. We identified 485 potential references through initial screening, then screened titles and abstracts, reviewed 262 full texts, and ultimately identified 30 core studies that met the inclusion criteria. To assess the quality of each article, we used the Newcastle-Ottawa Scale, the Cochrane Risk of Bias Tool, and AMSTAR-2.

**Results:**

Evidence indicates that a widespread epidemic of male loneliness is absent, although specific groups are at higher risk. Younger males (ages 18–29) appear to be experiencing a sharp increase in loneliness, while older males (ages 60+) show more stable patterns. However, this trend is accompanied by a notable imbalance in the evidence base, with younger men underrepresented relative to older populations. Traditional masculine norms, relationship loss, unemployment, disabilities, and veteran status have all been identified as risk factors for male loneliness. Our research demonstrated that male loneliness is associated with serious health problems, including depression, cardiovascular disease, cognitive decline, risk of suicide, and increased mortality. While community-based programs targeted toward males show high participation, they are almost entirely under-evaluated.

**Conclusion:**

Male loneliness is a patterned, life-course-related experience and should therefore be viewed as an ongoing process rather than a single “epidemic” event. Existing intervention strategies do not sufficiently consider how socialized gender processes influence men’s social withdrawal or disengagement. Future research may include conducting male-only randomized controlled clinical trials and longitudinal studies.

**Systematic review registration:**

https://osf.io/q97yv/, The review protocol was developed a priori and has been made publicly available through the Open Science Framework (OSF) to enhance transparency and reproducibility.

## 1 Introduction

Loneliness has emerged as a prominent object of public, political, and scholarly concern in recent years, frequently framed as a widespread social pathology with profound implications for population health and social cohesion. In the United States and other high-income countries, loneliness is increasingly described as an “epidemic,” invoked to explain rising mental health problems, premature mortality, and the perceived erosion of social life (1, 2). These framings were amplified by the COVID-19 pandemic, which intensified social disruption, altered everyday routines of contact, and renewed attention to loneliness and social isolation across the life course (3, 4). Yet as loneliness research has grown rapidly in fields such as epidemiology, psychology, sociology, and public health, its growth has also produced a fragmented evidence base, in which researchers from various disciplines use divergent concepts, definitions, and analytical approaches to measure loneliness (1, 2, 5). This lack of a unified conceptual framework complicates efforts to compare findings and draw consistent population-level inferences. However, in addition to conceptual inconsistency, loneliness research is methodologically fragmented; that is, how one defines, measures, and analyzes loneliness influences which mechanisms are deemed plausible or worthy of consideration for potential policy interventions (1, 2).

Within this expanding literature, men occupy a paradoxical position that is as empirical as it is interpretive. Popular discourse frequently portrays men as uniquely vulnerable to loneliness, linking male social disconnection to suicide, declining community participation, and shifting gender roles, yet the quantitative evidence has been less consistent about sex differences in loneliness prevalence (6, 7). However, meta-analytic analyses of studies across all stages of the life cycle typically find small or zero differences in average levels of loneliness by gender, with men sometimes reporting comparable or lower prevalence than women in later life (6, 7). Studies show that men appear to be at significantly greater risk for severe negative outcomes related to loneliness, such as suicide and premature death, compared to other groups, regardless of whether men report feeling lonely more or less than women (8). This discrepancy between the prevalence, lived experience, and the consequences associated with loneliness raises essential questions about the conceptualization, measurement, and interpretation of loneliness relative to gender and whether previous synthesis studies have captured the social processes through which loneliness results in adverse health outcomes for men (6, 7). Despite growing public concern, existing syntheses have not adequately distinguished between overall prevalence, subgroup vulnerability, and gendered mechanisms shaping men’s experiences of loneliness and social isolation. This review addresses that gap by providing a gender-informed, life-course synthesis of men in the United States, with particular attention to mechanisms, health consequences, and intervention alignment.

### 1.1 Purpose

The focus on the United States reflects both the availability of high-quality longitudinal and epidemiological data and the distinct structural context of U.S. healthcare systems, social policy frameworks, and gender norms. These factors influence how loneliness is experienced and reported, providing a U.S.-centered synthesis that is meaningful within a population-based perspective. Against this backdrop, there is a need for a systematic synthesis that foregrounds men not merely as a demographic subgroup, but as a socially situated population whose experiences of loneliness are shaped by gendered norms, life-course transitions, and structural conditions (9, 10). The present review responds to this need by synthesizing high-quality evidence, examining specific aspects including the prevalence and temporal trend data, as well as the life-course and gender-based risk factors, the health effects, and the effectiveness of interventions aimed at reducing loneliness (6, 8, 11, 12). The study has three major goals: First, it seeks to provide clarity about the patterns of loneliness that exist among men in various age groups and in different social environments, placing American data into the context of other high-income countries (6, 11). Second, the study will synthesize evidence related to the psychological and physical health effects of loneliness using longitudinal and meta-analysis findings to determine both the extent and consistency of these relationships (8, 13, 14); Third, the study examines the existing evidence on interventions aimed at reducing loneliness using a gender-informed approach, evaluating the level of correspondence between the interventions tested and the social reality of men’s lives (9, 12, 15). Overall, the study intends to advance beyond generalized claims about a “loneliness epidemic among men” to develop a detailed and evidence-based view of when, why, and to what degree loneliness emerges in men, and how such an understanding can inform future research and public policy directions (2, 5). Unlike prior reviews that primarily aggregate prevalence estimates, the present synthesis integrates epidemiologic trends with gendered social mechanisms and intervention responsiveness. By focusing on male-specific pathways across the life course, this review aims to move beyond descriptive comparisons toward a more mechanistic understanding of when and why loneliness becomes clinically and socially consequential for men.

## 2 Methodology

### 2.1 Review design and analytic orientation

This study was conducted as a systematic literature review following the Preferred Reporting Items for Systematic Reviews and Meta-Analysis (PRISMA) guidelines to enhance transparency, methodological rigor, and reproducibility of the evidence synthesis (16), with a focused analytic emphasis on loneliness among men in the United States. A protocol was developed *a priori* but was not prospectively registered. To enhance transparency, the protocol has been made publicly available via the Open Science Framework (OSF).^1^ The review was designed to balance comprehensive coverage of the loneliness literature with a focused gender-informed synthesis. Initially, the research strategy was expanded to encompass all available literature on loneliness; however, data were analyzed using a subset of studies that met established inclusion/exclusion criteria, demonstrated sufficient methodological rigor, and had interpretive value. While it can be argued that using the distinction between “retrieval” (i.e., identifying all possible sources) and “inclusion” (i.e., selecting sources for synthesis) provided a means of avoiding aggregation of heterogeneity in studies, this approach also provided a clear representation of how the selection process occurred. Analytic sufficiency of the review results was not based on the total number of included studies, but on the quality of the evidence (e.g., prevalence/trends, risk factors, health impacts, and interventions) that cohered to support each respective domain. Therefore, inclusion of additional studies that do not include specific information regarding male-identified aspects, or that lack methodologic transparency, will not impact the review’s results. Lastly, while the review focuses on men in the United States, evidence from other high-income Western countries has also been incorporated to provide a reference point for prevalence rates, measurement approaches, and health associations observed in settings with social and health care infrastructures comparable to the United States.

In this review, loneliness is defined as the subjective distress arising from a perceived gap between desired and actual social relationships, whereas social isolation refers to the objective absence or limited frequency of social contact. These constructs are analytically distinct and are treated as such throughout this synthesis. Measures of loneliness varied widely, including single-item items such as “How lonely do you feel?” (17) and the UCLA Loneliness Scale and its many variations (18). Measurement variability is viewed as an important aspect of the study of loneliness rather than as a methodological error that could have been eliminated by *post-hoc* statistical adjustments to create comparable measures across studies. No new pooling of quantitative data was conducted for this review. Instead, the authors selected and interpreted pooled estimates of outcomes derived from existing meta-analyses in their original context(s). Wherever previous meta-analyses reported outcome estimates for combined exposure constructs (i.e., loneliness and/or social isolation), this was considered a limitation of the underlying evidence base rather than a conceptual conflation arising from the current review.

### 2.2 Search strategy and data sources

A comprehensive search strategy was implemented across four bibliographic databases—PubMed/ MEDLINE, CINAHL, PsycINFO, and Scopus to capture peer-reviewed empirical studies. Searches were also conducted in Google Scholar to identify additional eligible studies that may have been missed by the four database queries. Google Scholar search results were ranked by relevance and reviewed sequentially until “saturation” was reached, defined as the absence of new eligible studies on consecutive pages (20 results per page). This structured stopping rule was applied to enhance reproducibility and minimize selection bias. Although this strategy enhanced coverage of potentially relevant studies, reliance on relevance-ranked results and saturation criteria introduces limits to reproducibility inherent to Google Scholar searches; however, this strategy has been used in prior systematic reviews to supplement database retrieval where indexing variability exists (19, 20). This resulted in 485 records prior to removing duplicates. Following the removal of duplicate records (*n* = 185), 300 unique records remained that were screened based on titles and abstracts. To prevent premature exclusion of potentially relevant studies, conservative inclusion/exclusion criteria were applied during the initial screening phase. At this point, studies were excluded for any of the following reasons: if they did not focus on loneliness or a closely related construct; if the study was conducted solely outside of the USA and did not relate to U.S.-specific populations; if the study was not peer reviewed; or if the publication language was not English. Searches were conducted between June 2025 and December 2025, with the final search conducted on the 7th of December 2025.

Search terms were developed iteratively and combined concepts related to: (1) loneliness and social isolation; (2) men, males, and gender; (3) the United States and comparable high-income countries; (4) prevalence, trends, and epidemiology; (5) health outcomes and consequences; and (6) interventions and programs. Boolean operators (AND, OR) were used to combine terms within and across concept groups. Example search string:

(“loneliness” OR “social isolation” OR “social disconnection”) AND(“men” OR “male” OR “masculine” OR “gender differences”) AND (“United States” OR “USA” OR “America” OR “high-income countries”) AND (“prevalence” OR “trends” OR “epidemiology” OR “health outcomes” OR “interventions”)

Complete database search strategies are provided in Supplementary Appendix A.

### 2.3 Inclusion and exclusion criteria

#### 2.3.1 Inclusion criteria

- Empirical quantitative or qualitative studies addressing loneliness or social isolation among adult men (age 18+)
- Systematic reviews and meta-analyses synthesizing evidence on loneliness prevalence, determinants,
- consequences, or interventions
- Studies conducted in the United States or comparable high-income Western countries with extractable male-specific data
- Longitudinal cohort studies, cross-sectional surveys, randomized controlled trials, and quasi-experimental designs
- Research articles written in English and published in a reputable scientific journal.

#### 2.3.2 Exclusion criteria

- Case reports, editorials, and commentaries without original data or systematic synthesis
- Small convenience samples (*n* < 50) without clear generalizability
- Studies without validated measures of loneliness or social isolation
- Studies reporting only aggregate results without gender-disaggregated data
- Studies focused exclusively on children or adolescents (age < 18).

### 2.4 Time frame and emphasis

**Figure 1:**
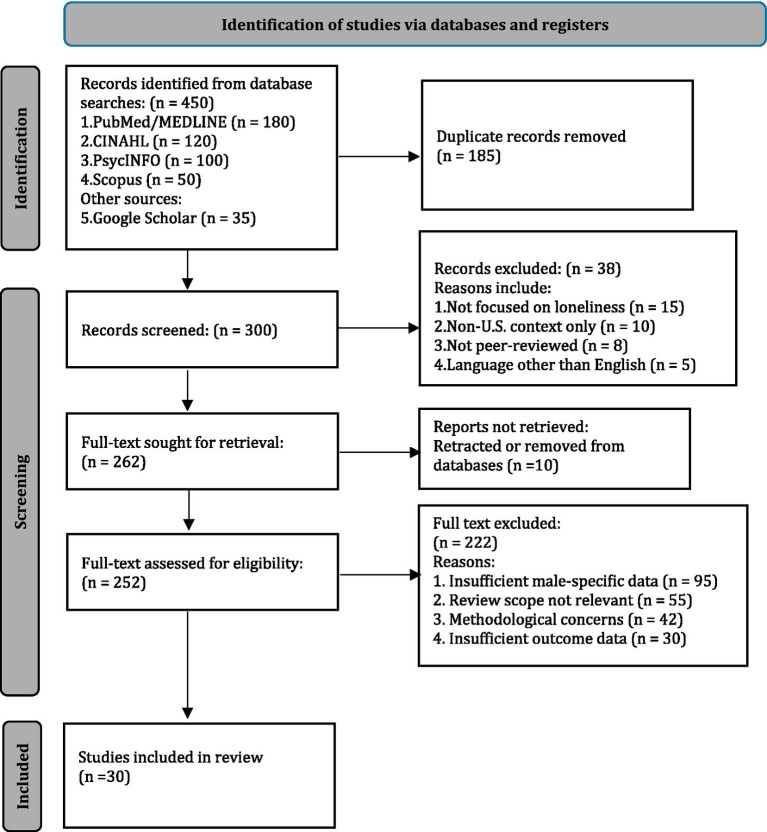
PRISMA flow diagram illustrating study identification, screening, eligibility, and inclusion.

While there was no firm cutoff date for publications, the focus was on articles from the past 10 years (2014–2025) that provided the most up-to-date information on current trends, current intervention trial results, and changes related to the COVID-19 pandemic. In addition, high-quality systematic reviews and key empirical studies published prior to 2014 were included, when applicable, as comparative measures to provide historical context and/or as a foundation for the literature on this topic (Figure 1).

### 2.5 Eligibility assessment and study selection

A full-text review was conducted of 300 articles that met the initial screening criteria, selected from an initial search of 485 records after removing 185 duplicates. Following title and abstract screening, 262 records proceeded to full-text assessment. Of these, 10 records were excluded prior to eligibility assessment due to retraction or removal from the source database, leaving 252 studies to proceed to full eligibility evaluation. These studies were then assessed against the following eligibility criteria: (a) whether the study addressed loneliness (subjective experience) or social isolation (objective social conditions) as the primary focus; (b) whether the study included male-specific data or gender-stratified analyses or could be theoretically applicable to the social experiences of men; (c) whether the study provided sufficient information about its methodology to facilitate interpretation of results; and (d) whether the study reported outcome data relevant to prevalence, risk factors, health outcomes, or interventions.

While 252 studies met general eligibility criteria, a subset of 30 studies were included in the primary analytic synthesis. To enhance transparency and consistency, studies were included in this core set if they met at least three of the following criteria: (1) reported male-specific or sex-stratified results; (2) employed longitudinal, meta-analytic, or randomized designs; (3) used validated measures of loneliness; and (4) provided effect estimates or sufficiently detailed outcome data to support mechanistic interpretation. Studies that did not meet these thresholds were retained in the broader narrative synthesis to provide contextual depth but were excluded from the primary analytic tables due to heterogeneity in study design, measurement, and reporting. A typology of study designs, populations, and thematic foci across all 252 studies is provided in Supplementary Table S1.

### 2.6 Synthesis approach

Given the heterogeneity of study designs, populations, and outcomes, synthesis combined narrative and quantitative approaches:

- *Quantitative synthesis*: Where multiple high-quality meta-analyses were available (e.g., for loneliness-health outcome associations), we reported pooled effect estimates with confidence intervals. We did not conduct original meta-analyses but rather synthesized findings from existing systematic reviews and meta-analyses. Consequently, the review necessarily inherits the conceptual definitions, exposure categorizations, and analytic decisions of prior authors, including instances where loneliness and social isolation were combined within outcome estimates, limiting causal and conceptual specificity.
- *Narrative synthesis*: For domains with greater heterogeneity or predominantly qualitative evidence (e.g., masculinity norms, help-seeking barriers), we employed structured narrative synthesis to identify themes, patterns, and evidence gaps.

### 2.7 Analytic corpus and evidence organization

The studies evaluated were heterogeneous in design, ranging from nationally representative cross-sectional surveys to longitudinal cohort studies, systematic reviews and meta-analyses, umbrella reviews, qualitative and mixed-methods syntheses, and intervention studies. This heterogeneous design enabled multilevel synthesis spanning epidemiologic patterns, life-course risk factors, health effects, and intervention effectiveness. While the review highlights the U.S. experience, evidence from other high-income countries was included to contextualize U.S. findings, identify gaps in available male-specific data, and triangulate the estimated effects using structurally comparable social, economic, and health systems. A master evidence table (Table 1) was constructed to map the characteristics of each study included in this analysis, which included study aims, design, population, methods, and principal findings. This table provided a linkage between the authors’ assertions and the evidence upon which they rested. The results of the studies were evaluated across four inter-related domains: (1) prevalence and trend over time of loneliness; (2) risk factors of loneliness across the sexes/life-course; (3) relationships between loneliness and multiple types of mental and physical health conditions; and (4) the efficacy of programs intended to either reduce or mitigate loneliness, focusing on males and aligned to gender.

**Table 1:** Evidence table for systematic literature review on male loneliness epidemic in the US.

#	Authors & year	Article title	Study Aim/objective	Study design	Sample size	Population	Key methodology	Primary outcomes/findings	Relevance to male loneliness
Male-stratified primary studies									
1	Kuwert et al. (2014) (28)	*Loneliness among older veterans in the United States: Results from the National Health and Resilience in Veterans Study*	Examine prevalence and demographic, military, health, and psychosocial correlates of loneliness among older U.S. veterans	Cross-sectional, nationally representative survey	*N* = 2,025 veterans aged ≥60	Older U.S. veterans (mixed-sex sample; veterans predominantly male, but sex-stratified loneliness estimates not reported in abstract)	Adapted Revised UCLA Loneliness Scale; multivariable analyses of demographic, military, health, and psychosocial correlates	44% reported feeling lonely at least some of the time; 10.4% often lonely. Strongest correlates: low perceived social support, insecure attachment, depressive symptoms, PTSD	Highly relevant to men because older U.S. veteran populations are predominantly male; provides U.S. prevalence and correlates, though loneliness estimates are not explicitly male-stratified
2	Rovito et al. (2022) (31)	*Social-Ecological Correlates of Loneliness Among Young Adult U.S. Males*	Identify intrapersonal, interpersonal, community, and societal correlates of loneliness among young U.S. men	Cross-sectional electronic survey with hierarchical regression	*N* = 495	U.S. men aged 18–25	Composite loneliness measure; hierarchical regression across social-ecological levels	Mental health diagnosis (*β* = 1.06), childhood physical/emotional abuse (*β* = 0.21), and childhood sexual abuse (*β* = 0.30) significantly associated with greater loneliness	Direct male-only evidence identifying early-life adversity and mental health as key pathways into loneliness among young men
3	Keum et al. (2021) (32)	*Distress Disclosure, Feeling Understood, Loneliness, and Psychological Distress Among Men*	Test whether loneliness mediates the association between distress disclosure, feeling understood, and psychological distress	Cross-sectional quantitative study	*N* = 1,827	Adult men recruited via an online men’s mental health platform (HeadsUpGuys)	Validated self-report measures; serial mediation path analysis with bootstrapped CIs; post-hoc multi-group analysis	Distress disclosure predicted lower distress directly (*β* = −0.116) and indirectly; loneliness was a significant mediator (*β* = −0.141), accounting for 64% of total effect; feeling understood alone was not	Provides rare male-only quantitative pathway evidence specifying loneliness as a mechanism linking masculinity-shaped disclosure to mental health outcomes
4	Kottke et al. (2025) (35)	*Feasibility of Community-Funding for Peer-Led Support for Men Who Struggle with Loneliness*	Describe and assess feasibility of a community-funded, peer-led support model for men	Program description/feasibility evaluation	Service data: >1,600 men; ~125,000 peer-support hours	Men engaged in a U.S. peer-led support organization	Aggregated service statistics; descriptive evaluation of activities and referrals	Demonstrated sustained reach, engagement, and clinician referral (~70%), indicating feasibility of peer-led male-focused support	Directly targets men; provides pragmatic evidence on scalability and engagement of male-aligned loneliness interventions
Prevalence & trends studies									
5	Buecker et al. (2021) (21)	*Is Loneliness in Emerging Adults Increasing over Time? A Preregistered Cross-Temporal Meta-Analysis and Systematic Review*	Evaluate temporal trends in loneliness among emerging adults from 1976–2019 using UCLA Loneliness Scale data	Cross-temporal meta-analysis and systematic review	449 means from 345 studies, 437 independent samples, total *N* = 124,855 emerging adults	Emerging adults (typically late teens to twenties) across multiple countries and decades	Preregistered cross-temporal meta-analysis of mean loneliness scores over calendar years using UCLA scale and regression of mean on year	Loneliness increased linearly across calendar years (*β* = 0.224, 95% CI [0.138, 0.309]); the increase corresponds to 0.56 standard deviations on the UCLA scale from 1976–2019	Indicates cohort-level increases affecting young men, though sex-specific trends require study-level data
6	Chawla et al. (2021) (22)	*Prevalence of loneliness amongst older people in high-income countries: A systematic review and meta-analysis*	Produce an evidence-based pooled estimate of loneliness prevalence among older adults (≥60 years) in high-income countries	Systematic review and random-effects meta-analysis	39 studies reporting data on ~120,000 older people; 31 studies included in meta-analysis	Older adults aged ≥60 years across 29 countries in high-income settings	Systematic search of five databases; pooled prevalence via random-effects meta-analysis and subgroup analyses by age and region	Pooled prevalence of loneliness 28.5% (95% CI 23.9–33.2%); moderate loneliness 25.9% (95% CI 21.6–30.3%); severe loneliness 7.9% (95% CI 4.8–11.6%)	Provides benchmark prevalence contextualizing older men’s loneliness
7	Surkalim et al. (2023) (25)	*Have Middle-Aged and Older Americans Become Lonelier? 20-Year Trends From the Health and Retirement Study*	Assess temporal trends (1996–2018) in episodic and sustained loneliness and subgroup differences in Americans ≥50 years	Longitudinal analysis of HRS cohort Waves 3–14	Analytic samples ranged *N* = 18,841–23,227 across waves	Middle-aged and older Americans (≥50 years) across sociodemographic subgroups by sex, race/ethnicity, cohort, education, employment, marital/living status	Lagged mixed-effects Poisson regression models for episodic and sustained loneliness; multivariate mixed models for predictors	Episodic loneliness declined from 20.1 to 15.5% and sustained loneliness from 4.6 to 3.6% over 1996–2018; males, Caucasians, partnered and non-living-alone individuals reported lower loneliness	Demonstrates declining late-life loneliness among men, countering epidemic claims
8	Surkalim et al. (2022) (11)	*The prevalence of loneliness across 113 countries: systematic review and meta-analysis*	Map global prevalence data and pooled prevalence estimates of loneliness across countries and age groups (2000–2019)	Systematic review and meta-analysis of nationally representative studies	57 studies provided data for 113 countries/territories; 212 estimates for 106 countries included in meta-analyses subset	Adolescents (12–17), young adults (18–29), middle-aged (30–59), older adults (≥60); geographic coverage heavily Europe for adults	Searches in multiple databases plus grey literature; random effects meta-analysis stratified by instrument, age group, and WHO region	Pooled adolescent prevalence ranged 9.2 to 14.4% by region; adult pooled analyses limited to Europe with lowest prevalence in northern Europe and highest in eastern Europe (e.g., older adults 21.3% in eastern Europe)	Provides global context for U.S. male findings
9	Hajek et al. (2024) (6)	*Chronic loneliness and chronic social isolation among older adults. A systematic review, meta-analysis and meta-regression*	Estimate prevalence of chronic loneliness among older adults and examine antecedents/consequences and heterogeneity sources	Systematic review and meta-analysis with meta-regression	17 studies in meta-analysis	Older adults (various cohorts)	Search across four databases; pooled prevalence estimation and meta-regression to explore heterogeneity	Estimated prevalence of chronic loneliness 20.8% (95% CI 16.1–25.5%); women 21.7% (95% CI 16.1–27.4%) and men 16.3% (95% CI 10.6–21.9%); single-item measures associated with lower prevalence estimates	Provides rare sex-stratified prevalence benchmarks
10	Hutten et al. (2021) (24)	*Risk Factors of Loneliness Across the Life Span*	Investigate a broad range of risk factors for loneliness using a large Dutch cross-sectional survey	Cross-sectional population survey	*N* = 52,341 respondents across late adolescence to old age	Dutch population spanning late adolescence to older adulthood; demographic and psychosocial variables included	Cross-sectional survey administered by regional public health services; multivariable analyses of risk factors by life stage	Risk factors across lifespan included being male, lower education, financial inadequacy, mental health problems, caregiving burden, and limited social contact; life stage specific risk sets identified	Demonstrates male risk in some population contexts
Health outcome studies									
11	Valtorta et al. (2016) (14)	*Loneliness and social isolation as risk factors for coronary heart disease and stroke: systematic review and meta-analysis of longitudinal observational studies*	Examine associations between loneliness/social isolation and incident CHD and stroke in longitudinal studies	Systematic review and meta-analysis of longitudinal observational studies	23 papers from 16 longitudinal datasets reporting 4,628 CHD and 3,002 stroke events	Longitudinal cohorts in high-income countries; age ranges varied across studies	Systematic search across 16 databases and pooling via random effects models; quality component approach	Poor social relationships associated with 29% increased risk of incident CHD (pooled RR = 1.29, 95% CI 1.04–1.59) and 32% increased risk of stroke (pooled RR = 1.32, 95% CI 1.04–1.68); subgroup analyses did not find gender differences	Indicates loneliness/social isolation have tangible cardiovascular risks for both sexes; absence of gender differential in pooled effects suggests relevance for men’s health outcomes.
12	Mann et al. (2022) (13)	*Loneliness and the onset of new mental health problems in the general population: a systematic review*	Synthesize longitudinal studies on whether baseline loneliness predicts new onset mental health problems	Systematic review and meta-analysis of longitudinal studies	20 studies included; meta-analysis of 8 independent cohorts for depression outcome	General population cohorts (majority older adults) across included studies	Systematic search of six databases plus grey literature; pooled adjusted ORs for new onset depression and narrative synthesis for other outcomes.	Pooled adjusted OR for new onset depression among adults often lonely vs. not often lonely = 2.33 (95% CI 1.62–3.34).	Demonstrates loneliness as a prospective risk for depression with implications for men’s mental health; many included cohorts were older adults but effect is generalizable across ages.
13	Wang et al. (2023) (8)	*A systematic review and meta-analysis of 90 cohort studies of social isolation, loneliness and mortality*	Quantify associations between social isolation/loneliness and mortality across cohort studies	Meta-analysis of longitudinal cohort studies	90 cohort studies including 2,205,199 individuals	Cohort studies spanning various populations and countries; sex-specific associations examined in subgroup analyses	Systematic search and pooling of longitudinal estimates for mortality outcomes; subgroup analyses including by sex	Social isolation and loneliness were associated with higher all-cause mortality (isolation RR ≈ 1.32; loneliness RR ≈ 1.14); social isolation also increased cardiovascular mortality (RR ≈ 1.34) and cancer mortality (RR ≈ 1.24), with elevated all-cause mortality among socially isolated individuals with cardiovascular disease (RR ≈ 1.28) and cancer (RR ≈ 1.51).	Confirms mortality risk associated with loneliness applicable to men; indicates sex-specific analyses were performed, making findings relevant for male mortality risk from loneliness.
14	Park et al. (2020) (36)	*The Effect of Loneliness on Distinct Health Outcomes: A Comprehensive Review and Meta-Analysis*	Evaluate loneliness effects across multiple health outcomes and identify risk factors/mechanisms and interventions	Systematic review and meta-analysis	114 studies included in qualitative review (number used in meta-analyses varies by outcome)	Studies across age groups and settings; proportion male varies by outcome/study	Reviewed studies using UCLA or de Jong Gierveld loneliness scales; meta-analytic pooling by outcome domains	Found medium to large effects of loneliness on health outcomes, largest for mental health and wellbeing; observed a gender effect for cognition associations (more pronounced with higher proportion male)	Indicates loneliness is linked to adverse health outcomes and flags gender-specific patterns (cognition) that may be relevant to understanding male loneliness consequences
15	Su et al. (2022) (4)	*Prevalence of loneliness and social isolation among older adults during the COVID-19 pandemic: A systematic review and meta-analysis*	Provide pooled prevalence estimates of loneliness and social isolation among older adults during COVID-19 pandemic	Systematic review and meta-analysis	30 studies including 28,050 participants	Older adults across studies conducted during COVID-19 pandemic (various countries)	Searches EMBASE, PsycINFO, Medline, Web of Science for studies Jan 2000–Nov 2021 limited to pandemic period; pooled prevalence estimation	Pooled period prevalence of loneliness among older adults 28.6% (95% CI 22.9–35.0%); pooled social isolation 31.2% (95% CI 20.2–44.9%); prevalence higher in studies conducted >3 months after pandemic start	Relevant to male loneliness by documenting high and increasing prevalence of loneliness among older adults during COVID-19 (≈29%), a group in which older men are at elevated risk due to narrower, role-dependent social networks; lack of sex-disaggregated estimates highlights persistent gaps in male-specific evidence.
Intervention studies									
16	Ratcliffe et al. (2020) (9)	*Men and loneliness in the ‘west’: A critical interpretive synthesis*	Critically summarizes research substantively related to men and loneliness in Western high-income nations	Critical interpretive synthesis (qualitative and quantitative studies)	79 studies met inclusion criteria	Studies from Western Europe, North America, Australasia; included qualitative and quantitative research focused on men/gender and loneliness	Predefined search across seven databases; synthesis produced seven synthetic constructs characterizing men’s loneliness	Seven constructs: men’s loneliness tied to social networks; emotional reticence; romantic relationships importance; measurement differences by sex; risky behavior links; masculinity insufficiency; intersectional identity influences	Directly focused on men; provides gendered theoretical constructs and implications for male-targeted interventions and measurement considerations.
17	Maes et al. (2019) (7)	*Gender differences in loneliness across the lifespan: A meta–analysis*	Quantify gender differences in loneliness and developmental period variations across lifespan	Meta-analysis	399,798 individuals (45.56% male), contributing 751 effect sizes	Participants across lifespan and multiple countries including South America and Europe	Meta-analytic aggregation of studies reporting sex/gender differences in loneliness across developmental periods	Across the lifespan, gender differences in loneliness were negligible (overall Hedges’ *g* = 0.07), indicating similar mean levels of loneliness among males and females. Moderator analyses showed small but significant variation by age, sampling scope, and year of publication.	Directly addresses gender differences, providing context for male loneliness patterns across life stages though specific effect sizes not reported in snippet
18	Zagic et al. (2021) (12)	*Interventions to improve social connections: a systematic review and meta-analysis*	Compare effectiveness of interventions on objective social contact and perceived quality of social connections in adults	Systematic review and meta-analysis of RCTs	58 controlled trials involving 8,780 participants, of whom 30.8% are males	Adult participants (mean age ~62); mixed clinical and community samples primarily in high-income countries	Systematic searches of Medline, Embase, PsycINFO, PubMed; pooled effect sizes (Hedges’ g) using random effects models	Interventions improved objective social contact (Hedges’ *g* = 0.43) and perceived quality (Hedges’ *g* = −0.33); increasing access to people most effective for objective contact (*g* = 0.67); psychological interventions best for perceived quality (*g* = −0.53)	Provides evidence on intervention types likely to improve social contact and perceived connection; limited male-specific reporting but informs approaches applicable to men
19	Hansen et al. (2024) (32)	*Tackling social disconnection: an umbrella review of RCT-based interventions targeting social isolation and loneliness*	Synthesize and appraise systematic reviews of RCTs on interventions for social isolation and loneliness	Umbrella review of systematic reviews of RCTs	29 systematic reviews included (16 with meta-analysis, 13 narrative syntheses)	Reviews covered young people, all ages, and older adults with varied geographic focus	Searches across multiple databases up to June 2023; quality appraisal with AMSTAR2; synthesis of SR findings and subgroup effects	Found small-to-moderate beneficial effects overall; social interventions helped social isolation and psychological interventions helped loneliness but effects were small and heterogeneous	Synthesizes RCT evidence applicable to male populations; highlights small effect sizes and need for targeted research on subgroup effects including males.
20	Morrish et al. (2023) (28)	*What works in interventions targeting loneliness: a systematic review of intervention characteristics*	Identify intervention characteristics associated with effectiveness for loneliness reduction in general populations	Systematic review with narrative synthesis	22 intervention studies included; diverse age groups and formats	General population studies; included older adults and other age groups; gender reporting varied	Searches across 8 databases; critical appraisal with JBI and CASP; narrative cross-study comparison of intervention features.	14/22 studies effective in reducing loneliness; effective features included group formats, active participation, between-session interaction, tailored learning mechanisms; recommendation for longer follow-up and attention to male populations with lower educational levels	Notes importance of considering males and lower-education populations in future interventions; highlights format elements that may be effective for men.
21	Ma et al. (2020) (27)	*The effectiveness of interventions for reducing subjective and objective social isolation among people with mental health problems: a systematic review*	Evaluate RCT evidence for interventions targeting subjective/objective social isolation among people with mental health problems	Systematic review of randomized controlled trials	30 RCTs included (15 subjective isolation, 11 objective isolation, 4 both). *N =* 3,080.	People with mental health problems (clinical populations); participant characteristics varied across trials.	Searched three databases for RCTs; extracted intervention types and outcomes; narrative synthesis due to heterogeneity.	Significant results in a minority of trials; methodological limitations (small samples) limit conclusions; promising approaches include cognitive modification for subjective isolation and mixed strategies with supported socialization for objective isolation.	Relevant to men with mental health problems experiencing loneliness; indicates some targeted strategies that could be adapted for male clinical population.
22	Welch et al. (2024) (15)	*In-person interventions to reduce social isolation and loneliness: An evidence and gap map*	Map evidence on effects of in-person interventions across ages/settings and identify gaps	Evidence and gap map synthesizing primary studies and systematic reviews	513 articles included (421 primary studies and 92 systematic reviews)	All age groups; majority focused on older adults and high-income countries, especially US/UK/Australia	Extensive database search up to Feb 2022; coding in Eppi-Reviewer; AMSTAR2 for review quality; mapping by intervention type/delivery/outcome.	Most reviews rated critically low quality; evidence concentrated in interpersonal and community-based interventions; major gaps at societal/community policy levels and process/implementation reporting.	Highlights distribution of intervention evidence relevant to men (many studies in high-income countries including the US) and identifies gaps for male-targeted or policy-level interventions.
Comprehensive reviews									
23	Lim et al. (2020) (1)	*Understanding loneliness in the twenty-first century: an update on correlates, risk factors, and potential solutions*	Update literature on correlates, risk factors and propose a conceptual model to inform interventions	Invited narrative review/conceptual synthesis	Not applicable (review)	Broad across age groups and settings; multidisciplinary literature synthesis	Literature review since 2006; synthesis into a proposed conceptual model linking demography, health, socio-environmental factors.	Synthesizes post-2006 evidence to propose a multi-level conceptual model of loneliness, identifying demographic, health, biological, and socio-environmental correlates and mapping intervention pathways; does not generate pooled prevalence or effect estimates.	Interprets gendered and life-course risk factors; informs mechanisms and intervention targets that can be applied to male loneliness though not male-exclusive.
24	Rezaei et al. (2022) (2)	*Loneliness and health: An umbrella review*	Overview of systematic and meta-analytic studies on loneliness epidemiology, etiology, health associations, and interventions	Umbrella narrative review synthesizing multiple systematic reviews and meta-analyses on loneliness and health	No explicit inclusion count reported.	Reviews covered a range of populations and conditions (childhood, adulthood, elderly, clinical groups).	Systematic search across databases; AMSTAR-2 quality assessment; narrative synthesis of review findings.	Loneliness associated with poor well-being, increased risk of mental and physical health conditions; interventions mostly focused on older adults with none-to-moderate benefits and heterogeneity across studies.	Summarizes health risks and intervention evidence relevant to men; underscores need for population-specific research including male subgroups.
25	Gasull-Molinera et al. (2024) (5)	*The impact of loneliness on mental and physical health outcomes: An umbrella review*	Collate and grade evidence linking loneliness to adverse health outcomes across reviews	Umbrella review of systematic reviews/meta-analyses	13 systematic reviews (4 meta-analyses); no pooled participant sample size reported.	Reviews cover multiple populations and conditions across life course	Systematic search up to Aug 2023; AMSTAR-2 risk of bias assessment and narrative synthesis	Found associations between loneliness and poorer mental/physical health and suggested but not confirmed causal links; quality of reviews often low	Reinforces health impacts of loneliness applicable to men; underscores need for higher-quality, male-focused longitudinal research.
26	Dahlberg et al. (2021) (10)	*A systematic review of longitudinal risk factors for loneliness in older adults*	Identify longitudinal risk factors for loneliness in older adults to guide interventions.	Systematic review of longitudinal observational studies	34 longitudinal studies (from 7 cohorts); no pooled participant *N* reported	Older adults (various cohorts across studies).	Systematic search and synthesis of longitudinal associations between risk factors and later loneliness.	Consistent longitudinal risk factors for loneliness included partner loss or being unpartnered, limited social networks, low social activity, poor self-rated health, and depression.	Relevant to older men through risk factor identification (e.g., spousal loss, health declines), though male-specific effects not highlighted
27	Di Perna et al. (2022) (21)	*A Systematic Literature Review of Loneliness in Community Dwelling Older Adults*	Synthesize antecedents, consequences, and intervention evidence for loneliness among community-dwelling older adults (≥50) published 2016–2021	Systematic review using PRISMA	49 usable articles included (published 2016–2021)	Community-dwelling older adults aged 50 + across studies	PRISMA systematic search and narrative synthesis of antecedents, consequences, and intervention outcomes	Antecedents/consequences differ by loneliness type; interventions improving personal/psychosocial resources had better outcomes than technology/social connection alone; mixed intervention results	Relevant to older men in community settings; emphasizes psychosocial resource strengthening which may inform male-tailored interventions
28	Ernst et al. (2022) (3)	*Loneliness Before and During the COVID-19 Pandemic: A Systematic Review with Meta-Analysis*	Synthesize prospective studies comparing loneliness before and during the pandemic to assess changes.	Systematic review and meta-analysis of longitudinal and pseudo-longitudinal studies	34 studies; total *N* = 215,026 participants (longitudinal subset *N* = 45,734)	General and clinical populations across multiple countries and ages.	Extraction of summary data from longitudinal and pseudo-longitudinal studies; risk of bias assessed using ROBINS-I; random-effects meta-analyses calculating standardized mean differences (Hedges’ g) for loneliness scores and log odds ratios for loneliness prevalence	Loneliness increased during the pandemic relative to pre-pandemic periods with small effect sizes. Among longitudinal studies, loneliness scores increased (19 studies; SMD = 0.27, 95% CI 0.14–0.40), and loneliness prevalence increased (8 studies; logOR = 0.33, 95% CI 0.04–0.62). Results were robust across study designs, timing of assessments, and clinical versus non-clinical samples, with substantial heterogeneity observed	Provides population-level evidence of pandemic-related increases in loneliness; findings are not male-specific but contextualize temporal shifts that may differentially affect men and underscore the need for gender-sensitive analyses of risk and protective factors.
29	Nordin et al. (2024) (31)	*A Scoping Review of Masculinity Norms and Their Interplay with Loneliness and Social Connectedness Among Men in Western Societies*	Map and synthesize evidence on how masculinity norms shape loneliness and social connectedness among men	Scoping review	13 empirical studies (from 1,730 records screened)	Adult men in Western societies	JBI scoping review methodology; systematic database search; thematic synthesis	Traditional masculinity norms (e.g., self-reliance, emotional restraint) associated with increased loneliness and reduced help-seeking; social connection often mediated through activity-based ties; evidence largely qualitative/ observational; causal relationships not established	Provides focused insight into masculinity-linked loneliness among Western men; scope shaped by selective inclusion criteria and Western focus.
30	Bruce et al. (2019) (25)	*Loneliness in the United States: A 2018 National Panel Survey of Demographic, Structural, Cognitive, and Behavioral Characteristics*	Quantify loneliness and its correlates (including social media use) among U.S. adults	Cross-sectional national panel survey	*N* = 20,096	U.S. Adults (18+) in all 50 U.S. states	Cross-sectional survey using validated loneliness measures UCLA Loneliness Scale (Version 3): Standardized 20 item self-report, producing a total score from 20 to 80 (higher = more loneliness)	Social support and meaningful daily interactions had the strongest associations with lower loneliness. Social anxiety was most strongly associated with greater loneliness, followed by self-reported social media overuse and daily use of text-based social media Found that loneliness decreases with age.	National U.S. data can inform male loneliness prevalence and correlates.

### 2.8 Risk of bias assessment

Risk of bias was examined in each core analytic study using a design-specific tool. The quality of systematic reviews and meta-analyses was evaluated using the AMSTAR-2 criteria; longitudinal cohort and observational studies were assessed using the Newcastle–Ottawa Scale; and the quality of intervention studies was assessed using an adapted Cochrane Risk of Bias assessment framework. Confidence ratings were assigned overall as recommended by the published guidance rather than based on numerical scores. Cross-sectional survey studies were reviewed with attention to their sampling strategies, response rates, and measurement validity. In addition, studies that used valid, representative samples, measured loneliness, employed longitudinal designs, appropriately controlled for confounders, and experienced minimal attrition were given greater weight. Methodological limitations and heterogeneity are noted throughout the synthesis. Rather than serving as exclusion criteria, risk-of-bias assessments were used to contextualize findings and weigh the strength of evidence across domains. Although formal risk-of-bias tools were applied, confidence judgments are narrative and domain-specific rather than algorithmically derived, reflecting the heterogeneity of designs and outcomes across the analytic corpus. Detailed risk-of-bias assessments for all core analytic studies, including AMSTAR-2 evaluations for systematic reviews, Newcastle–Ottawa Scale assessments for observational studies, and adapted Cochrane criteria for intervention studies, are provided in Supplementary Tables S6–S8.

### 2.9 Data synthesis and reporting

Given the heterogeneity of study designs, populations, and outcome measures, a structured narrative synthesis approach was adopted. Summary Tables of mean differences between estimates (with confidence intervals), along with references to source material (large-cohort studies and meta-analysis), were provided to enhance transparency and support the nuanced interpretation of the study results. The demographic inclusion/exclusion of “men” in the evidence base was assessed at each stage of the synthesis process. Where relevant sex-stratified analyses existed, such data were referenced specifically; otherwise, when men were underrepresented due to the design of a particular dataset, or when men’s representation was obscured within a given dataset, the lack of representation was documented as another significant finding.

### 2.10 Author contributions and resolution of disagreements

The study selection and analytical synthesis involved the collaborative effort among the seven authors (DC, EC, SVW, JL, AK, EE & MS) who employed a multi-stage strategy at each of the levels in the systematic review. In each stage, there were particular roles established to facilitate consistency, rigor and transparency. Screening of title and abstracts was performed by an independent, parallel review of all records retrieved from the databases that had been searched. A total of 7 reviewers (EC, SVW, JL, AK, EE, MS, and DC) completed title and abstract screening, while DC completed all screening steps and reviewed every citation. Every citation was independently reviewed by DC and at least one s other reviewer (either EC, SVW, JL, AK, MS, or EE), and disagreements were discussed. All citations that had been assessed as “potentially eligible” for inclusion were brought forward for full-text assessment. This assessment was conducted by two reviewers for each citation, and DC was involved in every full-text review. DC and at least one additional reviewer evaluated full texts using criteria applicable to male participants, methodological issues, and outcome measures. Disagreements between reviewers regarding the evaluation of full texts were addressed via structured discussion; however, in some cases a third reviewer (SVW, JL, or MS), were consulted when necessary; although, most disagreements could be resolved before formally adjudication. Data extraction was an iterative process. DC and EC/SVW developed the initial template(s) based on previous guidelines, and DC extracted all of the primary data, which was then reviewed and confirmed by other reviewers.

When discrepancies arose, they were resolved by comparing and discussing the individual results. No unreported quantitative data were inferred. The analytic synthesis, which included developing pathway tables (i.e., risk factors, mechanisms, life-course position), was completed by DC in collaboration with all co-authors. Since many tables were theory-driven, categorization was considered provisional and refined during discussion. Final decisions were determined through consensus; DC ensured that all pathways and categories were consistent and coherent with the evidence presented and the narrative. Consensus was reached through discussion and ensured that methodologic rigor was maintained and that there was sufficient interpretive depth to assess the gendered mechanisms and life-course processes that required both qualitative judgments and quantitative assessments. Inter-rater reliability was calculated on a random subset of screened records. Agreement between reviewers was high (Cohen’s *κ* = 0.82 for title/abstract screening; *κ* = 0.87 for full-text review), indicating strong consistency in study selection decisions.

## 3 Results

### 3.1 Overview of included evidence

A total of 30 core analytic studies were included in the primary analysis in this SLR. Each section is reported based on the various themes the authors found throughout the articles to best address the broad spectrum of loneliness within the male experience, with a particular emphasis on high-income countries. Findings are synthesized with emphasis on a subset of core analytic studies (*n* = 30) selected based on predefined methodological and relevance criteria, alongside broader narrative integration of the full eligible evidence base. Table 1 summarizes the evidence found within the given articles and serves as the primary reference for the synthesis of information.

Multiple methodological studies, such as nationally representative cross-sectional surveys, large longitudinal cohort analyses, systematic reviews and meta-analyses, umbrella reviews, qualitative and mixed-methods syntheses, scoping reviews, narrative reviews, and intervention evaluations, were included in the results. The multiplicity of articles allowed for a synthesis of epidemiological patterns, life-course risk factors, health consequences, prevalence trends, temporal trends, and intervention effectiveness, all while displaying evidence gaps within the topic of male loneliness. The final screening assessed 252 articles for eligibility; however, 222 articles were excluded for either lacking male-specific data, possessing an irrelevant review scope, methodological concerns, or having insufficient outcome data. The given evidence was directed towards older individuals (>60) in high-income countries, with an evidence gap for younger (18–29) and middle-aged (30–59) adult males.

### 3.2 Prevalence and temporal trends in loneliness among men

As the corpus of 30 papers demonstrates, male loneliness varies within age, social position, and marital/ employment/ living situation (6, 21–26). Some studies have shown a decrease in loneliness historically overtime (22, 25) while one study shows us that loneliness increases by calendar years (*b* = 0.224, 95% CI [0.138, 0.309]) with 18–29 being the most prevalent time for loneliness (21), despite this, the term “loneliness epidemic” is stated to be an exaggeration across most articles (27). Pooled prevalence data from high-income countries revealed a 28.5% prevalence of loneliness, further indicating population trends (1, 11). Age-specific and subgroup data have been provided in Supplementary Table S2, which showcases the prevalence of loneliness within the age categorization of males, social standing, and environmental considerations.

Older U.S. populations (≥60) ~ 20–25% reported loneliness in a single-item/UCLA variant (6, 22), while older U.S. veterans (≥60) had a prevalence of 44% loneliness some of the time with 10.4% reporting being lonely most of the time as measured in the UCLA (28). Emerging adult men (18–29) reported a + 0.56 SD increase within the UCLA Loneliness Scale (21), while middle-aged men (40–59) possessed a stable decline within a single-item measurement (25). Episodic vs. sustained data were found for a general population sample of older men (≥50) with a 20.1% → 15.5% (1996–2018) episodic decrease present over time (25), pandemic-period older men (≥60) reported a prevalence of ~28–30% (3) in a mixed instruments measure. Lower levels of loneliness have also been reported within Northern Europe as compared to South and East Europe (25). A trend within a study showed that women, overall, felt lonelier when assessed by direct questions, whereas men were found to be more socially lonely (9). This raises questions about gender differences in the statistical data collected. A monolithic approach was taken in most articles on loneliness, treating gender as binary (21); this makes assessing temporal trends in male loneliness difficult. A dualistic approach to gender would be more beneficial for understanding the leading causes and prevalent trends within this population. These findings indicate that male loneliness is not uniformly elevated across the population but is concentrated within specific age groups and social contexts.

### 3.3 Gendered and life course risk factors for loneliness

Of the 30 articles reviewed, 14 examined life-course risk factors in the general population. Across these studies, loneliness was associated with lower education levels, financial strain, caregiving burden, limited social contact or support, disability, and mental health disorder diagnoses or symptoms (1–3, 10, 13, 15, 23–26, 28–31). Mental health factors, along with childhood adversity, are more strongly linked to loneliness among young men, indicating earlier developmental pathways and limited possibilities for preventive intervention. As detailed in Supplementary Table S3, eight traits were identified as male-specific risk factors for loneliness across the life course. Where male-specific effect estimates were unavailable, general-population meta-analytic data were included to contextualize potential health risks, with the absence of sex-disaggregated evidence treated as a substantive limitation of the literature rather than a methodological oversight.

Masculine gender norms as a risk factor after qualitative and survey data showed a link to emotional stoicism, often causing males to avoid disclosing distress and personal problems, leading to higher levels of loneliness in all life stages (32). Male social networks were more likely to be activity-based (centered around sports or work), characterized by low levels of emotional intimacy, and therefore more vulnerable to role transitions such as retirement (33). Two cohort studies identified relationship loss, including divorce and widowhood, as significant factors for loneliness in mid to late life due to the loss of a primary confidant (10, 25). These studies also linked early adversities such as childhood verbal and physical abuse to higher loneliness levels in young males, citing attachment disruption as the primary cause. Unemployment was also a risk factor for loneliness across all life stages, likely caused by shame and reduced participation in social gatherings (15, 23–25). Additional risk factors include living in a rural area, associated with isolation and limited access to services (15), as well as veteran status, where trauma and reintegration barriers were linked to lower social engagement and higher levels of loneliness (28).

Across studies, high prevalence rates of loneliness were reported for both men and women. A large meta-analysis found similar prevalence rates between men and women, however male-specific factors must be considered when analyzing this data (7). Variances related to gender norms (emotional stoicism, self-reliance, and discomfort with vulnerability) are a possible explanation for the stigmatizing nature of mental health, leading men to be less likely to seek mental health services and disclose accurate loneliness levels (32). This often leads men to rely heavily on work and spousal relationships, with retirement, widowhood, and functional decline identified as key predictors of incident loneliness among older adults (10). Interestingly, quantitative pathway analyses provided convergent evidence for these gendered mechanisms. In a large cross-sectional study (32) of adult men (*N* = 1,827), demonstrated that distress disclosure was associated with lower psychological distress both directly (*β* = −0.116, 99% CI [−0.177, −0.055]) and indirectly through loneliness. The total indirect effect accounted for 64% of the overall association (*β* = −0.206, 99% CI [−0.247, −0.165]), with loneliness emerging as a significant mediator (*β* = −0.141, 99% CI [−0.180, −0.101]), whereas feeling understood alone was not. A significant indirect serial effect through feeling understood and loneliness (*β* = −0.116, 99% CI − 0.177, −0.055) indicates partial serial mediation of loneliness as a central mediator in men’s disclosure processes and their psychological distress. The gendered and life-course risk factors identified through a structured synthesis of the included studies are shown in Supplementary Table S4.

### 3.4 Health consequences of loneliness

The studies reviewed consistently identified loneliness as a risk factor for both negative physical and mental health outcomes. Twenty-one out of 30 studies examined reported an association between loneliness and an increase in the risk of a variety of adverse outcomes including, including incident depression, cardiovascular disease and stroke, all-cause mortality, and cognitive decline or dementia (1, 2, 4–8, 12–15, 21, 23, 24, 28–31, 34–36). Rather than isolated findings, these results are observed across different study designs, age groups, and populations, suggesting that loneliness functions as a broad psychosocial stressor with cumulative effects on health. However, a critical limitation across the evidence base is the relative absence of male-specific meta-analytic estimates. Many reported effect sizes are derived from mixed-sex populations, requiring cautious interpretation when applied to men. However, this gap itself represents a key finding, highlighting the structural absence of gender-disaggregated synthesis in loneliness research and reinforcing the need for gender-informed analytic approaches.

Loneliness affects poor health outcomes through psychological and behavioral mechanisms. Multiple studies have demonstrated that participation in high-risk behaviors, such as tobacco smoking, alcohol and illicit drug use, are associated with loneliness in heterogenous populations (2, 5, 9). In particular, there is evidence that loneliness among older men is associated with increased rates of gambling and the utilization of commercial sex workers, which may worsen pre-existing risks to overall mental and physical health (2, 9).

These behavioral patterns are linked to an increased risk of suicide and suicidal ideation and self-harm (2, 5, 35). Chronic social disconnection has been linked to increased risk of mortality due to physiological pathways such as sleep disorders and decreased immune function, as documented in several studies (4, 29, 36). A substantial body of evidence shows that loneliness and social isolation are each independently linked to poorer cardiovascular health. However, many studies examine them together, noting associations with higher risks of hypertension and coronary heart disease, reflected in elevated body mass index, increased blood pressure, and higher heart rate (1, 4, 8, 14). A 2016 systematic review indicates that individuals with coronary heart disease have an increased risk of coronary heart disease as compared to those without, with a relative risk of 1.29 (CI: 1.04–1.59) (14) as demonstrated in Table 2.

**Table 2:** Effect estimates for health outcomes associated with loneliness.

Outcome	Effect estimate	95% CI	Study	Evidence strength
Incident depression	OR = 2.33	1.62–3.34	Mann et al. (2021) (13)	High (meta-analysis of longitudinal cohorts)
Coronary heart disease	RR = 1.29	1.04–1.59	Valtorta et al. (2016) (14)	High (meta-analysis of prospective studies)
Stroke	RR = 1.32	1.04–1.68	Valtorta et al. (2016) (14)	High (meta-analysis of prospective studies)
All-cause mortality (loneliness/social isolation)	OR range ≈ 1.26–1.50	Study-specific	Wang et al. (2023) (8)	High (umbrella review of cohort studies)
Cancer mortality (social isolation)	RR = 1.24	1.19–1.28	Wang et al. (2023) (8)	Moderate–High (meta-analytic synthesis)
Cancer mortality (loneliness)	RR = 1.09	1.01–1.17	Wang et al. (2023) (8)	Moderate (fewer pooled studies)
Cardiovascular mortality (social isolation)	RR = 1.34	1.25–1.44	Wang et al. (2023) (8)	High (meta-analytic synthesis)
Cognitive decline/dementia	Directional association reported; pooled estimate not consistent	Not pooled	Park et al. (2020) (34)	Moderate (heterogeneous evidence)
Sleep disturbance/quality of life	Directional association reported; pooled estimate not consistent	Not pooled	Park et al. (2020) (34), Gasull-Molinera et al. (2024) (5)	Moderate (heterogeneous outcomes)

Furthermore, individuals who report emotional isolation are at greater risk for experiencing mental health problems. As mental health problems have been shown to have negative effects on the body’s inflammatory response, the likelihood of developing coronary heart disease is increased (37). Maladaptive coping strategies, including poor nutrition, inadequate sleep and decreased physical activity, while worsening mental state, can also damage physiological responses and create a potentially damaging cycle that further increases the risk of developing coronary heart disease (37). Supplementary Table S3 consolidates these findings into six main domains of outcomes: incident depression, coronary heart disease, stroke, cognitive decline and dementia, suicide and suicidal ideation, and sleep disturbance, demonstrating the consistent nature of the associations across studies. Incident depression was identified as the most commonly reported outcome supporting its key role as both a consequence and potential driver of loneliness (5–7, 13, 15, 21, 23, 28, 29, 35, 36).

Several studies further indicate bi-directional relationships between neurocognitive effects, with loneliness increasing dementia risk and cognitive decline, while dementia itself increases risk for social disconnection and loneliness (2, 4, 10, 15, 36). Cardiovascular outcomes demonstrate similar patterns. Individuals experiencing both loneliness and social isolation showed a 29% increased risk of coronary heart disease and a 32% increased risk of stroke (1). Finally, suicide related outcomes appear closely related to stigma and social rejection, particularly among men, reinforcing associations between loneliness, psychological distress, and mortality risk (35). Collectively, the literature supports loneliness as a meaningful psychosocial risk factor for men, with consistent associations across mental, cardiovascular, and mortality outcomes.

### 3.5 Intervention evidence and male relevance

Ten of the 30 articles directly examined loneliness interventions, focusing on three areas: social (skills training, social access, and social support), psychological (psychotherapy, cognitive behavioral therapy, and mindfulness practice) and community based (Men’s sheds and peer led support groups) (2, 12, 15, 28–30, 33–36). Four additional studies lacked intervention-specific data but used their findings to inform future interventions. Mann et al. (13) and Wang et al. (8) suggest developing methods for health care workers to identify loneliness and refer patients to resources when necessary. Two articles proposed differing approaches in gender-based intervention: Ratcliffe et al. (9) suggested a gender-sensitive approach due to male-specific challenges in help seeking, while (7) suggested that interventions should be directed to both men and women with individualized intervention strategies centered around different types of loneliness.

As summarized in Table 3, interventions varied by target. Social-contact interventions aimed at increasing social connection yielded small to moderate effects (*g* = 0.43), while “access to people” interventions produced the largest social-contact effect (*g* = 0.67) (12, 29). Interventions targeting connection are typically tailored to increase contact, but not necessarily to reduce subjective loneliness. They are also summarized as having no male relevance; however, they are tailored to yield the strongest social-contact modality in the meta-analysis. Perceived connection quality interventions primarily target connection quality in conjunction with loneliness (*g* = −0.33) and have a smaller tailoring effect on perceived connection quality. Psychological interventions are noted to be the best-performing category within the integrated summary, with a target of loneliness and perceived connection quality (*g* = −0.53) and are tailored for CBT/cognitive approaches (12, 26, 36). Men’s sheds (male-aligned communities) foster communal participation within the male subgroup; no pooled estimate has been reported for this intervention category; however, there is a strong emphasis on male relevance and tailoring despite the lack of evidence (9, 33). Peer-led male groups and peer-support models primarily aim to support belonging and engagement; despite the lack of comparable data, this intervention is considered a highly relevant category.

**Table 3:** Interventions to reduce loneliness: effects and male relevance (integrated summary).

Study	Intervention type/approach	Primary target/outcome	Effect estimate (as reported)	Notes on male relevance/tailoring
Zagic et al. (2021) (13)	Meta-analysis of randomized trials, grouped by strategy: *improving social support*, *improving social skills*, *increasing social contact*, *addressing maladaptive social cognition*	Loneliness-related outcomes across trials (loneliness and allied social-connection measures; trial outcomes varied)	Pooled effect sizes (Hedges’ g): improving social support *g* = 0.21; improving social skills *g* = 0.47; increasing social contact *g* = 0.18; addressing maladaptive social cognition *g* = 0.53. By strategy: increasing social contact *g* = 0.43; addressing maladaptive cognition *g* = 0.53; improving social support *g* = 0.21; improving social skills *g* = 0.47. Sub-strategies reported: facilitating social contact/access to people *g* = 0.67; enhancing social support *g* = 0.22.	Not male-specific; typically mixed-sex trials with limited sex-stratified reporting. Useful as the core pooled-effect benchmark for “what works” overall, but male tailoring is not built into the evidence base.
Ma et al. (2020) (27)	Systematic review of RCTs in people with mental health problems (heterogeneous intervention types)	Primary outcomes included loneliness, perceived social support, and objective social isolation	No pooled effect size reported; the review reports significant effects in only a minority of trials and highlights methodological limitations (including small samples). “Preliminary evidence” is noted for cognitive modification (subjective isolation) and mixed strategies/supported socialization (objective isolation).	Not male-specific. Relevant mainly as intervention-evidence caution (heterogeneity; limited strong recommendations), and as a source for psychological/cognitive approaches that may be adaptable to men but are not evaluated as male-tailored.
Morrish et al. (2023) (28)	Systematic review of intervention characteristics (design features linked to effectiveness; varied delivery formats)	Loneliness measured as the primary outcome (validated scale or single item)	22 studies included: 14 effective in reducing loneliness; 5 unclear; 3 no decrease. Key features highlighted (not effect sizes): between-session interaction; clear learning mechanisms; active participation; opportunities for facilitator/group interaction; varied teaching/learning styles.	Not male-specific, but the authors explicitly flag that future interventions should consider men (and lower educational levels). Helps justify why “engagement design” matters (how interventions are structured, not only what they target).
Welch et al. (2024) (16)	Evidence & gap map of in-person interventions across ages/settings (maps where evidence exists; not an effect synthesis)	Mapping interventions targeting social isolation and/or loneliness	No pooled effects (its purpose is to map and make evidence discoverable). The background emphasizes inconsistent effectiveness findings in the broader intervention literature.	Not male-specific; useful to understand the intervention field is large but uneven, and to show where male-aligned approaches may be underrepresented in rigorous evaluations.
Di Perna et al. (2022) (21)	Systematic literature review of intervention studies among community-dwelling older adults	Loneliness (the review notes the field often treats loneliness generically despite multiple types—social/emotional/existential)	Mixed intervention results; interventions focused on improving personal and psychosocial resources “fared better” than interventions emphasizing technological and social connections alone. No pooled effect sizes reported.	Not male-specific; relevant as an older-adult intervention signal consistent with argument that psychosocial resource-strengthening may matter more than contact/tech alone—potentially important for older men.
Hansen et al. (2024) (32)	Umbrella review (reviews of loneliness interventions; includes systematic reviews and meta-analyses)	Loneliness intervention effectiveness (across intervention types)	No pooled effect sizes; it characterizes the evidence base as mixed/heterogeneous, with interpretive limits shaped by review/trial quality.	Not male-specific; supports discussion claim that even where interventions show promise, the evidence base is methodologically uneven, and male-specific inference is limited.
Kottke et al. (2025) (33)	Male-aligned peer-led community support model (Face It Foundation; peer groups + activities; feasibility/program description)	Men’s loneliness addressed through friendship/social connection building (complements psychotherapy)	Descriptive “reach” outcomes (not a loneliness-scale effect): >1,600 men supported; ~125,000 h of peer-led support; ~70% referred by a mental health professional.	Male-specific by design; strong engagement/implementation relevance, but abstract does not report standardized loneliness effect sizes or randomized evaluation—fits “high engagement, low evaluative rigor” tension.
Keum et al. (2021) (30)	Mechanism-focused pathway analysis (intervention-informing)	Psychological distress; loneliness as mediator of disclosure processes	Serial mediation: total indirect effect *β* = −0.206 (99% CI − 0.247 to −0.165), accounting for 64% of total association between distress disclosure and psychological distress; loneliness significant mediator *β* = −0.141 (99% CI − 0.180 to −0.101); feeling understood alone not significant.	Male-only sample. Provides rare quantitative evidence specifying loneliness as the key mechanism linking disclosure to mental health among men. Directly informs why interventions must reduce loneliness—not only encourage disclosure. Relevant for gender-sensitive intervention design rather than efficacy testing.

Psychological and cognitive-based interventions demonstrate small, pooled effects, with an umbrella review reporting a near-zero overall effect size (*g* = 0.07) (7). Subsequent reviews reported small but significant effects for selected psychological and educational interventions; other intervention types demonstrated non-significant pooled effects (34). Social cognition and expectation-based interventions, that targeted maladaptive social cognition and negative social expectations, have shown the largest percent reductions compared to all other types of interventions. Effect sizes are small and vary widely across studies; they have been inconsistently reported (12). Objective social isolation interventions produced relatively consistent improvements in measured social interaction, whereas perceived loneliness showed either smaller improvements than those for objective social isolation or no statistically significant effect (12, 29). Group-based interventions were also found to have a positive relationship with measurable objective social contact than those conducted on an individual basis, due to small sample sizes and the large amount of heterogeneity among studies; pooling the results and determining a reliable estimate was not possible (29, 30).

Mixed- and multi-component interventions did not demonstrate superior outcomes compared with single-component interventions. Additionally, many studies found no significant reduction in loneliness from baseline to post-intervention (30, 35). Sex-stratified quantitative intervention outcomes were largely unavailable. No pooled effect estimates for men were reported, limiting the assessment of differential effectiveness by sex (8, 13). One plausible explanation for the relatively strong engagement observed in male-aligned community programs is identity congruence. Activity-based and peer-led formats may reduce stigma associated with emotional disclosure while allowing men to maintain alignment with masculine role expectations. Future trials should explicitly test whether intervention effectiveness is mediated by perceived gender congruence, stigma reduction, or changes in network structure. Taken together, the intervention literature suggests a misalignment between what engages men (activity-based, identity-congruent programs) and what has been most rigorously evaluated (CBT and social-contact models). A detailed prioritization of these evidence gaps is provided in Supplementary Table S5.

## 4 Discussion

The current systematic review synthesized data from 30 studies with diverse objectives, populations, and methodologies to identify the scope, characteristics, causes, and consequences of loneliness among men. This synthesis challenged both alarmist claims of an overall “loneliness epidemic” that minimized men’s experiences of social isolation and loneliness. The review provided a more detailed view of loneliness in relation to age by integrating prevalence trends, risk factors, health consequences, and evidence on interventions. Furthermore, the review offered insights into possible ways to address growing concerns regarding loneliness among young American men, including whether effective interventions are available; the extent to which structural and psychosocial risk factors contribute to this problem; and the focus of future research. Overall, the results demonstrated that loneliness is not a single entity, but rather a complex, life-course experience shaped by gender-related norms, social roles, and structural factors (with clear observable mental and physical health implications). Importantly, the present findings do not minimize the public health relevance of loneliness. Rather, they refine its epidemiologic characterization among men by demonstrating that risk is unevenly distributed and strongly shaped by life-course and gendered mechanisms.

While this study focuses on male loneliness, it is situated within a broader gendered context of disconnection from society. According to prior research, women, transgender, and non-binary people are also lonely but they have gender-specific routes of loneliness and ways of responding to loneliness (38). In their systematic review and meta-analysis of the literature on loneliness, Chawla et al. (22) reported that, in six of nine studies, females were more likely than males to report loneliness. The remaining three studies did not find a statistically significant difference in loneliness by gender. Hajek et al. (6) reported a prevalence of 21.7% for chronic loneliness among females, whereas males reported 16.3% across 17 studies; however, male-specific factors must be considered when interpreting these data (7). As has been noted, men’s reticence about reporting emotional distress may lead to an underestimation of loneliness in males because indirect assessment methods indicate men report higher levels of loneliness than direct questioning (1, 38). This pattern may also reflect social desirability bias, whereby men are less likely to endorse explicitly stigmatized terms such as ‘loneliness’ in direct questioning, resulting in divergence between direct and indirect measurement approaches. When intersecting factors such as sexual orientation and race/ethnicity are considered, the relationships between sex/gender and loneliness become even more complex. For example, in a college-based sample that consisted of 7% transgender or non-binary students, 79% of those same students reported feeling lonely, which supports the need to consider the demographics of the population when examining the prevalence of loneliness (39).

Life-course evidence indicates that patterns of loneliness vary across age groups. International longitudinal and prevalence research indicates that while rates of both episodic and persistent loneliness have decreased by approximately 2% per year for men 50 years of age and older since 2000, men 50 + years of age continue to experience less chronic loneliness than do women of equivalent age in sex-stratified international meta-analyses (6, 25). Several subsets of men continue to be at increased risk of chronic loneliness, including those who are unpartnered, live alone, have a disability, and/or are U.S. veterans. The likelihood that veteran status increases the risk of loneliness is attributable to the combined effects of the institutional history of military service, the traumatic events they experienced, and the difficulty of reentering their pre-military civilian social networks (28). Some studies report higher levels of loneliness in non-veteran populations; however, these findings may reflect methodological flaws rather than the beneficial effects of veteran status (26).

In contrast, cross-temporal meta-analytic evidence demonstrates the increasing trend of loneliness for emerging adult males across 18–29 years of age from various studies conducted over several decades (21). Young male U.S. adults are at increased risk for developing a mental health diagnosis, have an increased history of adverse experiences during childhood and have limited interpersonal resources related to their degree of loneliness (31). However, a key finding of this review is the divergence between epidemiological trends and evidence availability: although younger men appear increasingly at risk, they remain underrepresented in the empirical literature, which continues to focus predominantly on older adult populations. This imbalance limits the strength of age-specific conclusions and highlights a critical gap in the current evidence base. The data also illustrate that individual vulnerability can be combined with societal structural factors such as delayed family formation, lower socioeconomic status, and reduced engagement in social activities (8, 13, 14, 36). The research findings presented above challenge the general notion that late life is the primary period during which individuals experience loneliness and demonstrate young adulthood is a high-risk period for loneliness among young males (24).

Evidence comparing pre- and post-COVID periods suggests that the pandemic intensified attention to loneliness without fundamentally altering its underlying distribution among men. While pandemic-period studies report short-term increases in loneliness, particularly among older adults and socially isolated populations (40, 41), longitudinal and cross-cohort evidence indicates that pre-existing risk factors—such as age, relationship status, health limitations, and social resources—remain the primary drivers of loneliness (10, 42). In this sense, COVID-19 appears to have amplified pre-existing vulnerabilities rather than producing a distinct epidemiologic shift in male loneliness (3, 43). Digital engagement is yet another aspect of loneliness among younger men, which is contested. Research suggests that college-aged Individuals who experience loneliness are at greater risk for engaging in computer-based entertainment and subsequently raises concern over the possible psychological effects of excessive internet use (44). The meta-analytic findings support the association between loneliness and internet addiction, and suggest that loneliness can serve as a predictor for compulsive online behaviors (8). It appears that younger Adults may be more susceptible to problematic internet use than their older counterparts, creating a generational divide in digital risk (8, 45). Post-COVID-19, excessive digital engagement throughout daily life domains has been identified as a possible risk factor for loneliness among younger adults (3).

A full understanding of male loneliness is based on how gender socialization and transitions across the life course are interrelated. Masculine identity norms (e.g., emotional restraint, self-sufficiency, and the devaluation of vulnerability) have been shown to limit emotional depth in friendships and shape network structures in ways that heighten vulnerability to loneliness (7, 9, 33). Although there have been few direct experimental tests of mechanisms of masculinity, the overall pattern of findings from observational research, qualitative descriptions of experiences of loneliness, and varying rates of participation in interventions provides support for these mechanisms’ contribution to understanding the development of loneliness among men. The nature of men’s friendships (i.e., friendships based on shared activities, routine contact, and limited emotional disclosure) and the reliance of these relationships on established social roles (e.g., work, marriage/partnership, good health) contribute to this increased vulnerability to loneliness (1, 9). Evidence obtained through longitudinal research suggests that retirement, loss of a spouse, physical or cognitive decline, and the onset of chronic illnesses experienced by a partner are all associated with the development of loneliness among older men. This longitudinal evidence further suggests that the emergence of loneliness often reflects the loss of supportive social structures (i.e., relationships), rather than the complete absence of supportive connections (10).

Studies such as (32), which link masculinity-based emotional mechanisms to loneliness and long-term mental health problems via quantitative pathway modeling, offer one of the few examples of a combination of qualitative mechanism studies and quantitative pathway modeling. Their findings indicate that emotional disclosure alone is insufficient to alleviate distress when no meaningful relationship has been established, as shown in Supplementary Table S3. This integration of both the qualitative narratives and the emerging quantitative mediation analyses provides confidence that gendered mechanisms exist that link masculinity norms, loneliness, and mental health issues. While the mechanisms identified by these studies will likely be important to clinicians beyond providing an explanation for why men may experience greater levels of loneliness and subsequent mental health issues, longitudinal meta-analysis also indicates that men report a significantly increased risk of experiencing incident depression after they report feeling lonely often (OR = 2.33, 95% CI 1.62–3.34) compared to women, even after adjusting for baseline mental health and demographic characteristics (13). Sex specific pooled estimates have been limited, but the overall pattern from both primary studies and qualitative research indicates that men are at greater risk than women due to a delay in seeking help and social stigma around expressing emotional distress (8, 32, 36). This same dynamic occurs in sexual minority men who experience both stigma and discrimination that limit their ability to disclose emotionally and seek out supportive relationships (32).

Physical health outcomes show consistent associations with both loneliness and social isolation. A meta-analysis found that social disconnection increased the risk for developing coronary heart disease (Relative Risk [RR] = 1.29; 95% Confidence Interval [CI]: 1.04–1.59), as well as stroke (RR = 1.32; 95% CI: 1.04–1.68) (14). The risk of all-cause mortality was also elevated and estimated to range from an odds ratio of approximately 1.26 to 1.50 (8). Although relative risks were similar for males and females, sex stratified analysis suggested that absolute mortality risk was greater for males which may be due to differences in health behaviors, seeking help and accumulated stress (8). There was considerable heterogeneity in evidence regarding other outcomes including cognitive decline/dementia, sleep disturbances, and quality of life (5, 35). Comparative summaries can be found in Supplementary Tables S9, S10.

The intervention literature (Table 3) is substantial but heterogeneous, with most approaches yielding small-to-moderate effects and limited attention to gender alignment. Meta-analyses indicate that, although there is evidence for modest improvements in social connections from social-contact interventions (*g* = 0.43), these findings have largely been limited to interventions that either actively facilitated social connections or provided support for accessing them (*g* = 0.67). Cognitive-behavioral psychological interventions have shown larger pooled effects on loneliness and perceived connection quality (*g* = 0.53) (12). However, heterogeneity in design, outcome measurement, and follow-up limits the ability to draw definitive conclusions (30, 34). Our research indicated that there is a lack of male specificity: most trials were gender neutral, reported on little or no sex-stratified results, and rarely addressed how masculinity-related factors may influence engagement and responsiveness (15). Male-aligned community programs, such as men’s sheds and peer-led, activity-based programs, demonstrate high levels of participant engagement and perceived meaningfulness. These types of programs are, however, rarely represented in rigorous randomized controlled studies. This disparity highlights a long-standing conflict between engaging older adult males in interventions and ensuring the validity of those interventions through rigorous evaluation (9, 35, 46, 47).

Furthermore, there is a significant lack of knowledge about how social norms for boys/young men impact engagement with their mental health. So, from a policy perspective, these findings indicate potential ineffectiveness of implementing a one-size-fits-all approach broadly categorized as “universal interventions.” On the other hand, male-specific interventions that lack rigorous methodology may also risk excluding themselves from developing evidence-based policy responses. Addressing the gaps in research identified here will require methodologically innovative approaches to evaluating male-focused prevention programs that use hybrid evaluation designs to assess relational/community-based interventions, while simultaneously implementing a preventive/life-course approach that addresses the issues of boys and young men before they become entrenched in patterns of loneliness. Research indicates that it is essential for families to provide healthy environments that support children’s development and help young people experience early validation of their ability to express emotions, thereby helping them remain open to discussing mental health throughout their lives (32, 48). The most significant factor that continues to prevent individuals from seeking help for their mental health is stigma, and the relationship between stigma, poor physical and mental health outcomes, and the barriers created by factors such as age, gender, culture, which limit an individual’s willingness to report on their own mental health (49, 50).

Overall, there is substantial evidence that men’s loneliness is an important social issue that has health implications and lacks effective interventions. This study has provided an initial framework for developing effective, credible, and equitable interventions for reducing the gap of knowledge between research and practice by analyzing patterns of epidemiology, life course development, and gender sensitive interpretation (6, 8, 9, 12–14, 25). In particular, strengths-based approaches that redefine traditional forms of masculinity that are consistent with relational involvement and seeking help may have the greatest potential for increasing men’s engagement in addressing their loneliness (47). From an implementation perspective, future work should move beyond efficacy trials toward hybrid effectiveness, implementation designs capable of evaluating engagement, reach, and sustainability of male-aligned interventions in real-world settings.

### 4.1 Strengths and limitations

The review has several strengths that distinguish it from previous syntheses of the loneliness literature. First, it combines a comprehensive identification of the evidence base with a deliberate selective analytic synthesis to provide depth in interpretation without sacrificing transparency. By focusing its analysis on a clearly defined core set of methodologically robust studies, the review avoids the common problem of narrative overgeneralization across heterogeneous and weak sources. Second, by emphasizing men as both a demographic category and a socially positioned population whose experience of loneliness is shaped by gendered normativity, life course transitions, and structural situations, this review provides a comprehensive and narrative examination of patterns of prevalence, health effects, and intervention efficacy for loneliness than typically provided in reviews focused on entire populations. Third, the combination of quantitative estimates derived from meta-analysis with longitudinal data about the life course of loneliness and qualitative data enhances the public health relevance of the findings and places loneliness in a continuum with other recognized risk factors for physical and mental illness.

There are several limitations to this systematic review that should be acknowledged. Although this review was conducted in accordance with PRISMA guidelines and formally assessed using the risk-of-bias tool, the analysis was intentionally limited to 30 core studies to balance breadth and depth in the analytical process. While selectively focusing on 30 core studies enhances interpretive rigor, it excludes literature that met broader eligibility criteria but lacked male-specific data, sufficient methodological transparency, or outcomes that could be extracted. The distinction between comprehensive retrieval and selective analytic synthesis necessitates interpretive judgment regarding male relevance and analytic contribution. Though these judgments were guided by predefined criteria and documented transparently through the PRISMA flow diagram and evidence tables, this review prioritizes interpretability over strict reproducibility, which may limit exact replication of the analytic corpus. Moreover, while the review adhered to PRISMA guidance for systematic identification and reporting, the analytic strategy intentionally combined systematic review methods with structured narrative and interpretive synthesis.

As such, the review does not aim to exhaustively aggregate all eligible studies, but rather to integrate convergent evidence across domains in a gender- and life course–informed manner. Additionally, although most of the identified studies are from high-income countries, most focus are on older adults. This limits the generalizability of the findings to younger men, men who are members of racial and ethnic minority groups, and men who experience multiple layers of social disadvantages. In addition, although evidence from other high-income Western countries was incorporated to contextualize U.S. findings, differences in welfare systems, labor markets, and gender norms may limit the direct transferability of prevalence estimates and intervention effects to U.S. populations. There may also be measurement of variability across studies that assess loneliness, particularly when the assessment of loneliness is based upon the self-report of individuals. Self-report result in a differential report of loneliness by sex, which may underestimate the prevalence of loneliness among men due to stigma and/or norms related to emotional independence/self-sufficiency. Although the review acknowledges the importance of intersectional factors such as race, class, and sexual orientation, the available literature rarely provides sufficiently stratified data to support systematic intersectional analysis among men, leaving within-male heterogeneity underexplored. Lastly, there is a dearth of male only/ male-stratified randomized controlled trial interventions that restricts causal inference about what interventions will work best for men, underscoring the need for more targeted evaluation.

### 4.2 Implications for policy and practice

The findings of this study have significant implications for public health policy and practice. First, the robust correlations between loneliness and poor physical and mental health clearly support the idea that loneliness is a legitimate area of public health concern, and not just an adjunct social issue. Given effect sizes comparable to established behavioral risk factors, screening for loneliness, and assessing the degree of loneliness would be appropriate as part of routine health care (especially in primary care, mental health services, and settings where there is a high proportion of older men and other groups at higher risk). Effective implementation of such interventions will require developing gender-informed strategies that account for how men experience, report, and disclose their feelings about loneliness differently. Second, the poor success of many current intervention strategies suggests that “one-size-fits-all” approaches to reducing loneliness are inadequate. Interventions that focus solely on increasing social interaction are unlikely to yield sustained benefits for men because they do not address men’s subjective experiences or account for gendered obstacles to social participation. On the other hand, high levels of engagement observed in community-based initiatives designed specifically for men suggest that alignment with the identity, preferences, and social norms of men is crucial-even if these programs do not fit well within traditional evaluation models. Therefore, public health policy frameworks that narrowly define acceptable evidence may inadvertently marginalize community-based social interventions most likely to reach socially isolated men. As such, supporting innovation in evaluation methodologies (in addition to continued funding for community-based social infrastructure) may be key to developing effective, evidence-based public health policies and practices to reduce the negative consequences of loneliness.

### 4.3 Future research directions

Future research on loneliness among men should prioritize several interconnected areas. Most notably, there is an urgent need for male-specific, male-stratified trials that assess not only the efficacy of an intervention, but its engagement, acceptability, and feasibility in real-world settings. Such studies should explicitly engage with gender norms and life-course transitions, rather than treating men as a residual category within mixed-sex samples. Longitudinal studies of boys and young men across transition points in their development and social lives will provide much-needed data to understand how loneliness trajectories unfold and to identify opportunities for early prevention. Furthermore, there is an urgent need to develop intersectionality informed research. As mentioned earlier in this discussion, very little has been written about how intersectional differences (race/ethnicity, socioeconomic status, geographic location, disability, sexual orientation) affect men’s loneliness. Future research needs to improve measures of loneliness among males by developing and validating gender-specific measures to reduce reporting bias and assess relational quality. Finally, implementation-focused research is necessary to determine which evidence-based loneliness-reduction interventions can be adapted and sustained across diverse community settings.

## 5 Conclusion

This systematic review demonstrates that loneliness among men is a patterned and consequential social phenomenon rather than a diffuse or exaggerated concern (51). Although research shows that fewer men than women feel lonely, the health risks for lonely men tend to be more severe than for women. Therefore, it is important to look beyond reported rates of loneliness to determine whether a social problem is relevant to public health. To develop effective interventions grounded in the social contexts in which men live, it is essential to understand how social expectations, life-course transitions, and related factors influence men’s risk of experiencing loneliness. A gender-informed approach to integrating the results of epidemiologic, health outcome, and intervention studies can help identify both when men experience loneliness and the most effective ways to address its consequences. Developing an effective strategy for addressing loneliness among men, therefore, requires more than simply increased awareness of the problem; rather, it demands methodologically sophisticated approaches, clearly defined concepts, and a long-term commitment to developing interventions that account for the social context of men’s lives.

## Data Availability

The original contributions presented in the study are included in the article/Supplementary material, further inquiries can be directed to the corresponding authors.

## Footnotes

1
https://osf.io/q97yv/overview?view_only=7de7fc979f38445b9fbdbe2a81ab456d
