# Supplementary material for: Loneliness without an epidemic: gendered pathways, health consequences, and intervention gaps among men in the United States
Source: Front Public Health. 2026 Jun 2;14:1817065. doi: 10.3389/fpubh.2026.1817065 (PMC13270468; doi:10.3389/fpubh.2026.1817065)
Supplement: Supplementary file 1 [file Supplementary_File_1.pdf]

Supplementary Tables

Supplementary Table S1. Typology of Full-Text Evidence Included in the Review (n = 252)

Evidence Category	Study Design	Number of Studies	Primary Contribution
Prevalence & Trends	Cross-sectional surveys	~85	Point prevalence, demographic gradients
	Longitudinal cohorts	~42	Temporal trends, life-course trajectories
Health Outcomes	Longitudinal cohort studies	~48	Associations with mortality, CVD, mental health
	Meta-analyses / umbrella reviews	~18	Pooled effect sizes, outcome robustness
Risk Factors	Quantitative observational	~32	SES, masculinity norms, life transitions
	Qualitative studies	~21	Gender norms, help-seeking barriers
Interventions	RCTs / quasi-experimental	~39	Intervention effectiveness
	Program evaluations	~12	Feasibility, implementation insights
Theory / Measurement	Conceptual / validation studies	~15	Measurement heterogeneity, theory
Total		252	

Supplementary Table S2. Prevalence of Loneliness in Men by Age Group and Context

Population	Age Group	Measure	Prevalence / Trend	Key Source(s)
Older adults (U.S., nationally representative)	≥50	Episodic loneliness (lonely in 1 wave) and sustained loneliness (lonely in 3 consecutive waves), Health and Retirement Study	Episodic loneliness declined 20.1% (1996) → 15.5% (2018); sustained loneliness declined 4.6% → 3.6% over the same period. Trends broadly similar across subgroups; men reported lower episodic and sustained loneliness than women.	Surkalim et al., (2023) [25]
Older adults (multi-country, chronic loneliness meta-analysis; sex-stratified)	Older adults (study-defined)	Chronic loneliness (enduring/persistent over time; operationalized within included observational studies)	Pooled prevalence of chronic loneliness: men 16.3% (95% CI 10.6–21.9%); women 21.7% (95% CI 16.1–27.4%); overall 20.8% (95% CI 16.1–25.5%).	Hajek et al., (2024) [6]
Older adults (high-income countries; general prevalence meta-analysis)	60+ (with subgroup estimates for 65–75; >75)	Loneliness prevalence (severity pooled where reported; instrument varies across included studies)	Pooled prevalence 28.5% (95% CI 23.9–33.2%); moderate 25.9% (95% CI 21.6–30.3%); severe 7.9% (95% CI 4.8–11.6%). Age subgroups: 65–75: 27.6% (95% CI 22.6–33.0%); >75: 31.3% (95% CI 21.0–42.7%) (no significant difference by older age group in pooled estimates). Note: these pooled prevalence estimates are not reported as male specific.	Chawla et al., (2021) [22]
Older U.S. veterans (nationally representative sample)	≥60	Loneliness questionnaire adapted from Revised UCLA Loneliness Scale	44% reported feeling lonely at least some of the time; 10.4% reported often feeling lonely.	Kuwert et al., (2014) [28]
Young adult U.S. men (male-only primary study)	18–25	Three-Item Loneliness Scale (Hughes et al., 2004); categorized loneliness frequency in analysis	46.3% experienced frequent loneliness in the sample (n=495).	Rovito et al., (2022) [31]
Older adults during COVID-19 (pandemic prevalence meta-analysis)	Older adults (study-defined)	Loneliness / social isolation prevalence during COVID-19	Pooled period prevalence: loneliness 28.6% (95% CI 22.9–35.0%); social isolation 31.2% (95% CI 20.2–44.9%). Prevalence higher in studies conducted >3 months after the pandemic start than within the first 3 months. Note: sex-disaggregated prevalence is not provided.	Su et al., (2022) [4]
Emerging adults (cross-temporal trend; not male-specific)	Emerging adulthood (study-defined)	UCLA Loneliness Scale scores (cross-temporal meta-analysis)	Secular increase in loneliness: +0.56 SD from 1976–2019 (not reported as male-specific).	Buecker et al., (2021) [21]

Supplementary Table S3. Health Outcomes Associated with Male Loneliness (Summary)

Outcome Domain	Effect Size (Pooled)	Study Type	Evidence Strength	Notes on Male-Specific Findings	Study
Mental health: incident depression	OR = 2.33	Meta-analysis of longitudinal studies	High	Pooled estimate is not reported as male-specific; supports strong longitudinal association between loneliness and later depression, but sex-stratified pooled effects are not consistently available.	Mann et al., (2021) [13]
Cardiovascular: incident coronary heart disease	RR = 1.29	Meta-analysis of longitudinal cohort studies	High	Pooled estimate not male-specific; nonetheless widely used as foundational evidence that loneliness/social isolation carry clinically meaningful CVD risk.	Valtorta et al., (2016) [14]
Cardiovascular: incident stroke	RR = 1.32	Meta-analysis of longitudinal cohort studies	High	Pooled estimate not male-specific; same interpretive caveat as above.	Valtorta et al., (2016) [14]
Mortality: all-cause (social isolation)	RR = 1.32	Meta-analysis of prospective cohorts (within umbrella review)	High	Umbrella/meta-analytic synthesis; sex-stratified patterns are not uniformly pooled; sex-stratified analyses in the literature and highlights elevated mortality risk with social disconnection.	Wang et al., (2023) [8]
Mortality: all-cause (loneliness)	RR = 1.14	Meta-analysis of prospective cohorts (within umbrella review)	High	Effect reported for loneliness as distinct from social isolation; not male-specific pooled estimate .	Wang et al., (2023) [8]
Mortality: cancer (social isolation)	RR = 1.24	Meta-analysis of prospective cohorts (within umbrella review)	Moderate–High	Reported at population level; not male-specific.	Wang et al., (2023) [8]
Mortality: cancer (loneliness)	RR = 1.09	Meta-analysis of prospective cohorts (within umbrella review)	Moderate	Smaller effect size; not male-specific; fewer pooled studies typically contribute to this estimate.	Wang et al., (2023) [8]
Mortality: CVD mortality (social isolation)	RR = 1.34	Meta-analysis of prospective cohorts (within umbrella review)	High	Reported for social isolation; not male-specific.	Wang et al., (2023) [8]
Mortality: all-cause among people with CVD (social isolation)	RR = 1.28	Meta-analysis of cohort studies in disease populations	Moderate	Disease-subgroup estimate; not male-specific.	Wang et al., (2023) [8]
Mortality: all-cause among people with breast cancer (social isolation)	RR = 1.51	Meta-analysis of cohort studies in disease populations	Moderate	Not male-relevant population-wise; included as part of the umbrella review's reported disease-subgroup mortality effects.	Wang et al., (2023) [8]
Cancer-specific mortality among people with breast cancer (social isolation)	RR = 1.33	Meta-analysis of cohort studies in disease populations	Moderate	Not male-relevant population-wise; included because it is explicitly reported in the same pooled evidence source.	Wang et al., (2023) [8]
Cognitive outcomes (cognitive decline/dementia)	Positive association reported; no pooled estimate reported in review	Narrative synthesis of systematic review evidence	Moderate	Some evidence suggests stronger cognitive impact in studies with larger proportions of men.	Park et al., (2020) [36]
Sleep disturbance / quality of life	Moderate positive association reported	Narrative synthesis of systematic review evidence	Moderate	Loneliness consistently associated with poorer sleep outcomes; sex-specific pooled effects limited.	Park et al., (2020) [36]; Gasull-Molinera et al., (2024) [5]
Psychological distress (mechanism-adjacent, male-only)	Not a pooled health-outcome effect; path coefficients reported	Male-only quantitative primary study (cross-sectional pathway analysis)	Moderate (mechanism evidence; not longitudinal)	Male-specific by design (N = 1,827); loneliness functions as a key mediator linking distress disclosure processes to psychological distress, supporting gendered mechanism plausibility rather than causal health-outcome inference.	Keum et al., (2021) [32]

Note: Where male-specific effect estimates were unavailable, high-quality general-population meta-analytic estimates were included to contextualize potential health risks. The absence of sex-disaggregated data is treated as a key limitation of the evidence base.

Supplementary Table S4. Male-Specific Risk Factors for Loneliness Across the Life Course

Domain	Risk Factor	Evidence Type	Life Stage	Mechanism	Study
Gender norms	Emotional stoicism / emotional restraint	Qualitative synthesis / interpretive	Adult–later life	Constrains disclosure and emotionally sustaining connection; narrows acceptable intimacy	Ratcliffe et al., (2020) [9]
Gender norms	Self-reliance / reluctance to seek help	Qualitative synthesis / interpretive	Adult–later life	Discourages help-seeking and relational reliance; reinforces solitary coping	Ratcliffe et al., (2020) [9]
Social networks	Activity-based friendships (vs. emotionally intimate ties)	Qualitative synthesis / interpretive	Adult	Produces ties that are situational and vulnerable to disruption; low emotional depth	Ratcliffe et al., (2020) [9]; Lim et al., (2020) [1]
Social networks	Reliance on work-based social ties	Qualitative synthesis / interpretive	Adult–midlife	Work organizes routine contact; disruptions remove socially sanctioned contexts for connection	Ratcliffe et al., (2020) [9]; Lim et al., (2020) [1]
Role transitions	Retirement	Longitudinal review synthesis	Older age	Loss of routine/work-based interaction; reduced structured opportunities for connection	Dahlberg et al., (2021) [10]
Role transitions	Unemployment / role loss	Qualitative synthesis / interpretive	Working age	Disrupts routine and identity-linked contexts where men maintain ties	Ratcliffe et al., (2020) [9]; Lim et al., (2020) [1]
Relationship loss	Divorce / widowhood / partner loss	Longitudinal review synthesis	Mid–late life	Loss of primary confidant; men's emotional support often concentrated in partner relationship	Dahlberg et al., (2021) [10]
Health decline	Functional decline / spousal illness	Longitudinal review synthesis	Older age	Shrinks mobility and participation; caregiving/health limitations reduce social activity	Dahlberg et al., (2021) [10]
Early adversity	Childhood physical/emotional/sexual abuse	Male-only cross-sectional primary study	Young adulthood	Long-run psychosocial vulnerability; linked to loneliness among young adult men	Rovito et al., (2022) [31]
Mental health	Mental health diagnosis / distress	Male-only cross-sectional primary study	Young adulthood	Psychological vulnerability intertwines with loneliness and constrained interpersonal resources	Rovito et al., (2022) [32]
Emotional processes	Lower distress disclosure	Male-only cross-sectional pathway analysis	Adult	Lower disclosure → less feeling understood → higher loneliness → higher psychological distress (serial mediation)	Keum et al., (2021) [32]
Psychosocial resources	Low perceived social support	Male-specific subgroup primary study (veterans)	Later life	Low support co-occurs with loneliness and mental health symptoms; reinforces isolation	Kuwert et al., (2014) [28]
Trauma / institutional history	Veteran status (older U.S. veterans)	Male-specific subgroup primary study	Later life	Trauma exposure and reintegration challenges co-occur with high loneliness prevalence	Kuwert et al., (2014) [28]
Socioeconomic position	Low education / financial strain	Systematic review synthesis across life span	All	Resource constraints reduce participation and social opportunity; associated with loneliness across stages	Hutten et al., 2021 [24]
Social contact	Limited social contact / small network	Systematic review synthesis across life span	All	Reduced interaction and network breadth associated with loneliness across life stages	Hutten et al., 2021 [24]
Care roles	Caregiving burden	Systematic review synthesis across life span	Adult–older age	Time/strain reduces social participation and increases vulnerability to loneliness	Hutten et al., 2021 [24]
Household context	Living alone / unpartnered status	Longitudinal U.S. cohort evidence (older adults)	Older age	Reduced daily companionship and support; higher loneliness risk in older populations	Surkalim et al., (2023) [25]

Supplementary Table S5. Priority Evidence Gaps Identified in the Review

Gap	Consequence	Research Priority	Study
Limited sex-disaggregated reporting in loneliness research	Weak male-specific inference despite large mixed-sex samples	Mandate routine sex-stratified analyses and reporting	Hajek et al., (2024) [6]; Surkalim et al., (2022) [11]; Welch et al., (2024) [15]
Overrepresentation of older adults; underrepresentation of young and midlife men	Risk of mischaracterizing loneliness as primarily a late-life issue	Expand longitudinal cohorts focused on adolescence → midlife transitions	Buecker et al., (2021) [21]; Rovito et al., (2022) [31]; Surkalim et al., (2023) [25]
Measurement heterogeneity (single-item vs validated scales; inconsistent thresholds)	Limited comparability of prevalence and effect estimates across studies	Harmonize use of validated loneliness instruments and reporting conventions	Chawla et al., (2021) [22]; Park et al., (2020) [36]; Hajek et al., (2024) [6]
Mechanisms linking masculinity norms to loneliness are under-tested quantitatively	Mechanisms remain inferred rather than specified, limiting intervention translation	Increase quantitative pathway and mediation analyses alongside qualitative work	Ratcliffe et al., (2020) [9]; Keum et al., (2021) [32]; Nordin et al., (2024) [33]
Scarcity of male-tailored intervention trials	Gender-neutral interventions may show limited uptake or relevance for men	Design and evaluate male-aligned interventions with sex-specific outcomes	Zagic et al., (2021) [12]; Welch et al., (2024) [15]; Morrish et al., (2023) [30]
High-engagement community programs lack rigorous evaluation	Promising male-focused programs remain marginal to evidence-based policy	Use pragmatic and hybrid effectiveness–implementation designs	Kottke et al., (2025) [35]; Ratcliffe et al., (2020) [9]
Conceptual overlap between loneliness and social isolation	Ambiguity in mechanisms and intervention targets	Explicitly distinguish and measure both constructs in study design	Valtorta et al., (2016) [14]; Wang et al., (2023) [8]; Park et al., (2020) [36]
Limited causal inference due to cross-sectional designs	Directionality and mediation pathways remain uncertain	Prioritize prospective, longitudinal, and quasi-experimental designs	Mann et al., (2021) [13]; Valtorta et al., (2016) [14]
Underrepresentation of marginalized male subgroups (race/ethnicity, sexuality)	Masked heterogeneity and limited equity relevance	Oversample and analyze intersectional male subpopulations	Rovito et al., (2022) [31]; Keum et al., (2021) [32]
Limited long-term follow-up and implementation data for interventions	Unclear durability, scalability, and real-world impact	Extend follow-up periods and report implementation outcomes	Morrish et al., (2023) [30]; Welch et al., (2024) [15]
Pandemic-era studies lack consistent sex-stratified trend analysis	Uncertain male-specific pandemic trajectories	Conduct sex-stratified longitudinal post-pandemic analyses	Ernst et al., (2022) [3]; Su et al., (2022) [4]

Supplementary Table S6. Risk of Bias Assessment: Systematic Reviews & Meta-Analyses (AMSTAR-2)

Study	Protocol / Registration	Search Adequacy	Risk of Bias Considered	Publication Bias Assessment	Overall Confidence (AMSTAR-2)
Chawla et al. (2021) [22]	PROSPERO registered	Comprehensive multi-database search	JBI critical appraisal tools	Not formally assessed	Moderate confidence
Hajek et al. (2024) [6]	Not reported	Multiple databases; meta-regression	Quality appraisal reported	Not formally assessed	Low confidence
Hansen et al. (2024) [34]	Not reported	Broad multi-database search	Explicit RoB assessment	Not reported	Low confidence
Mann et al. (2021) [13]	Registered protocol	Extensive multi-database search	Study quality assessed	Not reported	Moderate confidence
Maes et al. (2019) [7]	Not reported	Multiple major databases	No formal RoB tool specified	Funnel plot / bias checks reported	Low confidence
Park et al. (2020) [36]	Not reported	Multiple databases	Quality assessment reported	Not reported	Low confidence
Rezaei et al. (2022) [2]	Not reported	Multiple databases	AMSTAR-2 used for included reviews	Not applicable (umbrella review)	Low confidence
Su et al. (2022) [4]	Not reported	Multi-database pandemic search	Quality appraisal reported	Not reported	Low confidence
Surkalim et al. (2022) [11]	Registered protocol	Extensive multi-database search	Quality appraisal reported	Publication bias assessed	Moderate confidence
Valtorta et al. (2016) [14]	Not reported	Comprehensive multi-database search	Structured validity framework	Funnel plot reported	Moderate confidence
Wang et al. (2023) [8]	Not reported	Broad database coverage	Newcastle–Ottawa Scale	Funnel plots + Egger test	High confidence
Welch et al. (2024) [15]	Not reported	Comprehensive search strategy	AMSTAR-2 applied	Not reported	Moderate confidence
Zagic et al. (2021) [12]	Protocol referenced	Multi-database search	Explicit RoB assessment	Funnel plot + Egger test	High confidence
Morrish et al. (2023) [30]	Not reported	Broad multi-database search	Quality appraisal reported	Not reported	Low confidence
Gasull-Molinera et al. (2024) [5]	Not reported	Broad database search	Quality appraisal reported	Not reported	Low confidence

Supplementary Table S7. Risk of Bias Assessment: Longitudinal Cohort & Observational Studies (Newcastle–Ottawa Scale)

Study	Selection	Comparability	Outcome Assessment	Attrition	Overall Risk
Bruce et al., (2019) [26] (U.S. national panel survey; cross-sectional observational)	4/4	2/2	2/3	N/A (cross-sectional)	Low–Moderate
Rovito et al., (2022) [31] (young adult U.S. men; social media/online recruitment; cross-sectional observational)	2/4	1/2	2/3	N/A (cross-sectional)	Moderate–High
Keum et al., (2021) [32] (adult men; HeadsUpGuys online recruitment; cross-sectional observational; path analysis)	2/4	1/2	2/3	N/A (cross-sectional)	Moderate
Kuwert et al., (2014) [28] (National Health and Resilience in Veterans Study; U.S. veterans; cross-sectional observational)	4/4	2/2	2/3	N/A (cross-sectional)	Low–Moderate
Surkalim et al., (2023) [25] (Health and Retirement Study waves 1996–2018; longitudinal cohort analysis)	4/4	2/2	3/3	Low (weights updated for attrition; missingness rules described)	Low

Supplementary Table S8. Risk of Bias Assessment: Intervention Studies & Program Evaluations (Cochrane RoB / Adapted)

Study / Intervention Type	Randomization	Blinding	Attrition	Reporting Bias	Overall Risk	Primary Evidence Source(s)
CBT-based psychological interventions	Low	Moderate	Moderate	Low	Moderate	Zagic et al., (2021) [12]; Hansen et al., (2024) [34]; Morrish et al., (2023) [30]
Social contact / facilitation interventions	Moderate	High	Moderate	Moderate	High	Zagic et al., (2021) [12]; Welch et al., (2024) [15]
Men's sheds / activity-based male programs	Not randomized	Not feasible	Moderate	Moderate	High	Ratcliffe et al., (2020) [9]; Lim et al., (2020) [1]
Peer-led male support groups	Not randomized	Not feasible	Moderate	Moderate	High	Kottke et al., (2025) [35]; Ratcliffe et al., (2020) [9]

Supplementary Table S9. Contextualized Interpretation of Effect Sizes

Exposure–Outcome Relationship	Effect Size	Comparator	Contextual Interpretation	Study source
Loneliness → incident depression (new-onset)	OR = 2.33	Frequent loneliness vs not often lonely	More than doubled odds of developing depression over follow-up; magnitude is clinically meaningful for population mental health burden.	Mann et al., (2021) [13]
Loneliness/social isolation → incident coronary heart disease	RR = 1.29	Lonely/socially isolated vs not	~29% higher risk of CHD incidence; a moderate elevation comparable to many established psychosocial risk factors.	Valtorta et al., (2016) [14]
Loneliness/social isolation → incident stroke	RR = 1.32	Lonely/socially isolated vs not	~32% higher risk of stroke incidence; suggests social disconnection is a non-trivial vascular risk correlate.	Valtorta et al., (2016) [14]
Social isolation → all-cause mortality (general population)	Effect size = 1.32	Socially isolated vs not	~32% higher mortality risk; magnitude suggests clinically consequential exposure at population scale.	Wang et al., (2023) [8]
Loneliness → all-cause mortality (general population)	Effect size = 1.14	Lonely vs not	~14% higher mortality risk; smaller than isolation but still significant and meaningful given prevalence.	Wang et al., (2023) [8]
Social isolation → cardiovascular mortality	Effect size = 1.34	Socially isolated vs not	~34% higher risk of CVD mortality; supports cardiovascular relevance of objective disconnection.	Wang et al., (2023) [8]
Social isolation → cancer mortality (general population)	Effect size = 1.24	Socially isolated vs not	~24% higher cancer mortality risk; suggests downstream mechanisms may include access, support, and treatment adherence pathways.	Wang et al., (2023) [8]
Loneliness → cancer mortality (general population)	Effect size = 1.09	Lonely vs not	~9% higher risk; statistically significant but modest—interpret as small effect with potentially large population impact.	Wang et al., (2023) [8]
Social isolation (CVD patients) → all-cause mortality	Effect size = 1.28	Socially isolated vs not (within CVD samples)	~28% higher mortality risk among people already living with CVD; indicates heightened vulnerability under clinical risk.	Wang et al., (2023) [8]
Social isolation (breast cancer) → all-cause mortality	Effect size = 1.51	Socially isolated vs not (within breast cancer samples)	~51% higher mortality risk; large effect in a high-risk clinical population, plausibly reflecting support/treatment pathway sensitivity.	Wang et al., (2023) [8]
Social isolation (breast cancer) → cancer-specific mortality	Effect size = 1.33	Socially isolated vs not (within breast cancer samples)	~33% higher cancer-specific mortality; suggests isolation may matter for disease course or treatment continuity.	Wang et al., (2023) [8]
Distress disclosure → psychological distress (direct pathway)	β = −0.116	Higher vs lower disclosure (standardized path)	Small-to-moderate protective direct association: more disclosure corresponds to less distress (net of mediators/covariates).	Keum et al., (2021) [32]
Distress disclosure → psychological distress (indirect via loneliness)	β = −0.141	Higher vs lower disclosure (standardized indirect path)	Loneliness is the primary mediating mechanism: reduced loneliness accounts for a substantial portion of disclosure's association with lower distress.	Keum et al., (2021) [32]

Note: The "effect size" metric varies by study design (OR/RR/β) and is reproduced as reported, not harmonized, which is consistent with the narrative synthesis approach.

Supplementary Table S10. Evidence Strength by Domain (GRADE-Adjacent, Narrative)

Domain	Evidence Base	Consistency	Directness	Overall Confidence
Prevalence & temporal trends	Mix of meta-analyses/systematic reviews plus large U.S. longitudinal cohort evidence (e.g., cross-temporal synthesis; HRS trend analysis; pooled prevalence in older adults) Buecker et al., (2021) [21]; Chawla et al., (2021) [22]; Surkalim et al., (2023) [25]; Hajek et al., (2024) [6]	Moderate (estimates vary by measure, age, and context; trajectories differ by cohort/period)	Moderate (often not male-specific; sex-stratification inconsistent; measurement heterogeneity)	Moderate confidence
Gendered and life-course risk factors / mechanisms	Observational risk-factor syntheses + qualitative/mixed-method syntheses + limited pathway modeling in men Hutten et al., (2021) [24]; Dahlberg et al., (2021) [10]; Ratcliffe et al., (2020) [9]; Keum et al., (2021) [32]; Nordin et al., (2024) [33]	Moderate (convergent themes—masculinity norms, transitions, network structure—but quantitative tests uneven)	Moderate–High (mechanisms often inferred; limited male-only quantitative mediation evidence)	Moderate confidence
Mental health outcomes	Strong base of longitudinal cohort + meta-analytic evidence; pooled estimates for incident depression; broader reviews on mental health links Mann et al., (2021) [13]; Park et al., (2020) [36]; Wang et al., (2023) [8]	High (direction consistent; effect sizes stable across cohorts/reviews)	Moderate (sex-specific pooled effects rarely reported; male-specific vulnerability inferred rather than directly tested in meta-analyses)	High confidence
Cardiovascular outcomes	Meta-analysis of longitudinal cohorts for CHD/stroke + umbrella-level syntheses Valtorta et al., (2016) [14]; Wang et al., (2023) [8]; Park et al., (2020) [36]	High (effects consistently elevated)	Moderate (largely mixed-sex cohorts; limited male-stratified estimates)	High confidence
Mortality outcomes	Large-scale meta-analytic evidence including many cohorts, with isolation and loneliness examined separately; some sex-stratified interpretation reported Wang et al., (2023) [8]	High (robust across settings and populations)	Moderate (often not male-specific exposure–outcome estimates, but evidence base is large and prospective)	High confidence
COVID-era loneliness prevalence	Systematic review/meta-analysis of older adults + broader COVID loneliness syntheses Su et al., (2022) [4]; Ernst et al., (2022) [3]	Moderate (prevalence varies; higher later in pandemic)	Low–Moderate (older-adult focus; sex-disaggregated estimates limited; indirect relevance to men)	Moderate–Low confidence
Intervention effectiveness (general loneliness interventions)	Multiple systematic reviews/meta-analyses of interventions (CBT, social-contact, psychosocial), but heterogeneity is substantial Zagic et al., (2021) [12]; Welch et al., (2024) [15]; Hansen et al., (2024) [34]; Morrish et al., (2023) [30]	Moderate (small-to-moderate pooled effects; high heterogeneity)	Low–Moderate (mostly gender-neutral samples; limited sex-stratified outcomes; variable measures)	Moderate confidence
Male-aligned interventions (men's sheds / peer-led male groups)	Primarily qualitative syntheses and program feasibility/evaluation evidence rather than trial-grade outcomes Ratcliffe et al., (2020) [9]; Kottke et al., (2025) [35]	Moderate for engagement/acceptability; Low for quantified loneliness reduction	Moderate (high male relevance; but causal effects unclear)	Low confidence

Supplementary Appendix

Supplementary Appendix A: Full Database Search Strategies

Database	Date Searched	Filters Applied	Full Search Strategy
PubMed / MEDLINE	December 7, 2025	English; Humans	("Loneliness"[MeSH] OR loneliness[tiab] OR "social isolation"[MeSH] OR "social isolation"[tiab] OR "social disconnection"[tiab]) AND (men[tiab] OR male[tiab] OR masculinity[tiab] OR "gender differences"[tiab]) AND ("United States"[MeSH] OR "United States"[tiab] OR USA[tiab] OR America[tiab] OR "high-income countries"[tiab]) AND (prevalence[tiab] OR trends[tiab] OR epidemiology[tiab] OR "health outcomes"[tiab] OR interventions[tiab] OR programs[tiab])
PsycINFO	December 7, 2025	Peer-reviewed; English; Human	("loneliness" OR "social isolation" OR "social disconnection") AND ("men" OR "male" OR "masculinity" OR "gender differences") AND ("United States" OR "USA" OR "America" OR "high-income countries") AND ("prevalence" OR "trends" OR "epidemiology" OR "health outcomes" OR "interventions" OR "programs")
CINAHL	December 7, 2025	English; Peer-reviewed	("loneliness" OR "social isolation" OR "social disconnection") AND ("men" OR "male" OR "masculinity" OR "gender differences") AND ("United States" OR "USA" OR "America" OR "high-income countries") AND ("prevalence" OR "trends" OR "epidemiology" OR "health outcomes" OR "interventions")
Scopus	December 7, 2025	Article; English	TITLE-ABS-KEY ("loneliness" OR "social isolation" OR "social disconnection") AND TITLE-ABS-KEY ("men" OR "male" OR "masculinity" OR "gender differences") AND TITLE-ABS-KEY ("United States" OR "USA" OR "America" OR "high-income countries") AND TITLE-ABS-KEY ("prevalence" OR "trends" OR "epidemiology" OR "health outcomes" OR "interventions")
Google Scholar	December 7, 2025	English	Search combinations included: loneliness OR social isolation AND men OR male AND United States AND prevalence OR health outcomes OR interventions. Results were ordered by relevance and screened sequentially until saturation was reached (no new eligible studies identified across consecutive pages).
